# Exploring Excited-State Electronic Structure, Spectroscopy, and Nonadiabatic Dynamics with CP2K’s Multifaceted Approach

**DOI:** 10.1021/acs.jpca.5c02969

**Published:** 2025-08-04

**Authors:** Kota Hanasaki, Tjeerd Futaii de Jong, Konstantin Komarov, Ravi Kumar, Momir Mališ, Johann Mattiat, Luis Ignacio Hernandez-Segura, Lukas Schreder, Andrey Sinyavskiy, Sandra Luber

**Affiliations:** Department of Chemistry, 30964 University of Zurich, Winterthurerstrasse 190, 8057 Zürich, Switzerland

## Abstract

The CP2K software package provides a comprehensive suite of density functional theory-based methods for studying excited states and spectroscopic properties of molecular and periodic systems. In this review, we present recent developments and applications of several complementary approaches implemented in CP2K, including linear-response time-dependent (TD) and time-independent density functional perturbation theory (DFPT), delta self-consistent field (ΔSCF), and real-time TDDFT (RT-TDDFT). Nonadiabatic molecular dynamics (NAMD) capabilities are integrated with ΔSCF and TD-DFPT methods, in addition to Ehrenfest dynamics based on RT-TDDFT, enabling detailed investigations of photochemical processes and the excited-state dynamics in gas and condensed phase systems. Applications demonstrating the versatility of these methods include studies on solvated molecules, surface-bound photosensitizers, and two-dimensional materials. Spectroscopic methods encompass, e.g., ultraviolet–visible absorption, electronic circular dichroism, Raman (optical activity), infrared absorption, and vibrational circular dichroism spectra. We demonstrate that CP2K provides a unique and powerful toolkit for studying a wide range of excited-state phenomena in complex molecular and extended (periodic) systems.

## Graphical abstract

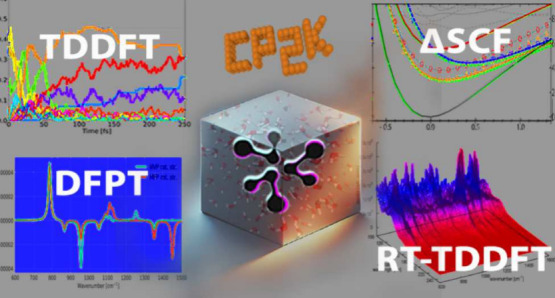

## Introduction

Accurate computational simulations are nowadays indispensable. Artificial photosynthesis for chemical production, photolithography for integrated circuits fabrication, and photodynamic therapy for cancer treatment are just a few examples where computational modeling of their underlying nonadiabatic processes plays a key role in their research and development. These simulations are challenging because, in addition to the system’s ground electronic state properties, excited electronic state properties must be explored. The latter have to be accurate as they determine the accuracy of the simulations. To achieve this, models have to be realistic, that is, as close as possible to the true system, so consequentially they should usually be large, as a proper inclusion of the environment into simulations is required. From a computational chemist’s perspective, there are still not many software packages well-suited for such tasks. Among them is CP2K, which offers a variety of implementations for excited-state electronic structure calculations.

The CP2K code is well-known in the community for its Gaussian and plane wave (GPW) method which has been widely applied. In computational chemistry, it has been used to study complex molecular and extended (periodic) systems. For example, in a recent study, Eliseeva et al. employed CP2K to investigate metal-involving halogen bonding in platinum­(II) complexes, demonstrating its utility for analyzing noncovalent interactions in organometallic compounds.^1^ In materials science, CP2K has, for instance, been applied to study the properties of bulk materials and surfaces. Yokelson et al. utilized CP2K for density functional theory (DFT)-based ab initio molecular dynamics (AIMD) simulations of a catalytic system, showcasing its performance on both CPU and GPU architectures.^2^

CP2K stands out as a software package capable of performing electronic structure-based AIMD simulations on systems containing many millions of atoms, a scale previously thought unattainable. This exceptional capability is achieved, among others, through the recent implementation of the nonorthogonal local submatrix method, a massively parallel algorithm that enables linear-scaling density functional theory calculations.^3^ Then this breakthrough has pushed the boundaries even further, enabling CP2K to achieve simulations of outstanding size and complexity, such as a SARS-CoV-2 spike protein system containing 83 million atoms.^4^ Regarding the accuracy and reliability of its DFT implementation, it has been the subject of extensive verification efforts. For example, Bosoni et al. have compared various DFT codes, including CP2K, against all-electron reference calculations for a diverse set of 960 crystal structures.^5^

However, all these studies were conducted in the ground electronic state. For excited electronic states, CP2K has its flavor of the Tamm–Dancoff approximation (TDA) of the time-dependent (TD) density functional perturbation theory (DFPT) (TD-DFPT) adapted to the GPW method and periodic boundary conditions (PBC).^6^ It has been mainly used for absorption spectra calculations in various systems,^7–15^ also for X-ray absorption spectroscopy.^124,258^ Its application for nonadiabatic molecular dynamics (NAMD) was limited,^16–18^ and, only after the analytical nuclear gradients became available,^19,20^ a full NAMD simulation at the TD-DFPT level of theory could be conducted.^21^ Similarly, the delta self-consistent field (ΔSCF) method was implemented in CP2K,^22^ and mostly applied for excited-state MD. Only after a different variation of the method was included, CP2K with ΔSCF was applied for NAMD simulation.^23,24^ The real-time (RT) TDDFT implementation in CP2K has been used for e.g. Ehrenfest molecular dynamics (EMD),^25,26^ and electronic density evolution for calculating voltage decay and transport property^27^ or stopping power.^28–30^ An O(N) scaling of EMD was demonstrated with subsystem linear-scaling implementation on systems with more than thousands of atoms, and an 30% acceleration when using GPUs.^25^ Spectroscopic application of RT-TDDFT included electronic circular dichroism,^31,32^ Raman spectroscopy,^33,34^ and Raman optical activity.^34^ RT-TDDFT was also extended to systems with the PBC in the Γ-point formulation to analyze Raman excitation profiles (including optical activity) in the liquid phase^33^ and solvent effects in optical spectra in the aqueous phase.^35,36^ For time-independent spectroscopy, DFPT was developed to compute Raman^37^ infrared (IR) absorption spectra,^38^ as well as NMR^39,40^ and EPR^40^ properties, both for periodic and nonperiodic systems. It was further extended to calculate vibrational circular dichroism (VCD) spectra for chiral molecules in a nonperiodic framework.^41,42^ Additionally, to achieve linear scaling and reduced memory requirements, DFPT-based computations of spectroscopic properties have been extended to an atomic-orbital-based linear response solver.^43–45^

In this review, we present an overview of the current excited-state capabilities of CP2K, with a focus on methodological developments and illustrative applications from the Luber group. We begin by describing the theoretical foundations, including the GPW approach to establish a solid foundation in the underlying DFT methodology. We then proceed to discuss the various excited-state methods available in CP2K. First, we explore the TD-DFPT approach, which enables the effective calculation of excited-state properties. Then the ΔSCF approach provides a straightforward way to optimize specific excited states. Further, we present applications for these methods in CP2K utilizing nonadiabatic molecular dynamics (NAMD) simulations with surface hopping algorithms to model excited-state dynamics and relaxation processes. Next, we show the DFPT formalism with the atomic orbital- and molecular orbital-based solvers to study, for example, Raman, Raman optical activity (ROA), infrared (IR) absorption, and vibrational circular dichroism (VCD) spectra. We then discuss the real-time TDDFT approach for calculating excited-state spectra of molecules and periodic systems, including optical absorption, electronic circular dichroism, (resonance-) Raman and ROA spectroscopy. Finally, we summarize the key features and capabilities of CP2K for excited-state calculations in the Conclusions.

## Methods

### DFT and GPW Formalism

Density functional theory (DFT) has become one of the most widely used methods for electronic structure calculations in condensed matter physics, materials science, and chemistry.^46,47^ To extend the applicability of DFT to larger and more complex systems, various computational approaches have been developed. One such approach is the GPW method, which combines the strengths of localized basis sets and plane wave expansions to represent the electron density.

The GPW method, as implemented in CP2K’s Quickstep module, offers an efficient approach for performing DFT calculations on large systems.^48^ At its core, the GPW method employs the Kohn–Sham DFT energy expression:

1 $$E\left[\rho \right]=E^{T}\left[\rho \right]+E^{V}\left[\rho \right]+E^{H}\left[\rho \right]+E^{\mathrm{xc}}\left[\rho \right]+E^{\mathrm{II}}$$

where *E* ^T^[ρ] is the electronic kinetic energy, *E* ^V^[ρ] is the electronic interaction with the ionic cores, *E* ^H^[ρ] is the electronic Hartree energy, *E* ^xc^[ρ] is the exchange–correlation energy, and *E* ^II^ represents the interaction energies of the ionic cores. For a more detailed description of the implementation of DFT within the GPW framework in CP2K, readers are referred to the original paper by VandeVondele et al.^48^

As noted above, the GPW method utilizes two representations of the electron density ρ.^49^ The first is based on an expansion in atom-centered, contracted Gaussian functions:

2 $$\rho \left(r\right)=\underset{\mu \nu }{\sum }D_{\mu \nu }\chi_{\mu }\left(r\right)\chi_{\nu }\left(r\right)$$

where *D*_*μν*_ is a density matrix element and χ_μ_(**r**) are contracted Gaussian functions. The second representation employs an auxiliary basis of plane waves:

3 $$\rho \left(r\right)=\frac{1}{\Omega }\underset{G}{\sum }\mathrm{\rho ̃}\left(G\right)\exp\left(iG\cdot r\right)$$

where Ω is the volume of the unit cell, **G** are the reciprocal lattice vectors, and i is the imaginary unit.

The transformation of the electron density between real and reciprocal space can be done by the Fourier transform:

4 ρ̃(G)=∫ρ(r)⁡exp(−iG·r)⁡dr

This interconversion between representations is the key advantage of the GPW method. In particular, the electron density, defined on the real-space grid, is efficiently transformed into reciprocal space using fast Fourier transforms (FFTs). This enables the rapid solution of the Poisson equation and allows evaluation of the Hartree potential in the plane-wave domain using mapping algorithms with near-linear scaling in system size, as detailed elsewhere.^48–51^

Additionally, the GPW method naturally incorporates PBC, making it well-suited for the simulation of both solids and liquids. The use of a plane wave basis allows for an accurate description of long-range electrostatic interactions.

To complete the description of the GPW method, it is important to note that the interaction between valence electrons and ionic cores is described using pseudopotentials, with CP2K implementing the Goedecker–Teter–Hutter (GTH) pseudopotentials.^52^

CP2K also offers the Gaussian and augmented plane wave (GAPW) formalism, an all-electron generalization of GPW. GAPW keeps the efficient plane-wave treatment for the smooth, long-range part of the charge density, yet augments it with atom-centered Gaussian contributions that exactly reconstruct the rapidly varying core density inside nonoverlapping atomic spheres. Because the hard (Gaussian) and soft (plane-wave) components are evaluated separately, the plane-wave cutoff can be kept moderate while the full Coulomb and exchange–correlation potentials still converge to all-electron accuracy, even for transition-metal or first-row elements where core states play a decisive role. The resulting energy expression contains inexpensive one-, two- and three-center Gaussian integrals plus a reduced FFT for the smooth part, so the overall scaling remains O(Nlog⁡N) with only a small prefactor compared to GPW.^48,50^

A broad variety of exchange–correlation functionals is available for evaluating the exchange–correlation energy, including local density approximations (LDA), generalized gradient approximations (GGA), meta-GGAs, and hybrid functionals incorporating exact Hartree–Fock exchange. While the native set of functionals in CP2K is limited, the LibXC interface significantly extends the range of available approximations, all of which are seamlessly supported within the CP2K framework.^53–56^

The exact Hartree–Fock exchange required by hybrid functionals involves the evaluation of four-center electron repulsion integrals (ERIs) over Gaussian basis functions. In CP2K, these integrals are computed analytically using the Libint library.^56,57^ To reduce the computational cost, CP2K also offers the auxiliary density matrix method (ADMM), which approximates the exchange calculation using a smaller auxiliary basis set. A GGA-based correction is applied to compensate the difference between the approximate and full densities.^58^ This approach yields speed-ups of up to 3 orders of magnitude in large systems while maintaining accuracy with errors below 1 kcal/mol, as demonstrated on the GMTKN24 benchmark.^58^

CP2K implements several important features that enhance its capabilities for large-scale DFT simulations. The code offers a hierarchy of increasingly accurate basis sets, from double-ζ to quadruple-ζ quality with polarization functions, allowing users to balance computational cost and accuracy.^59^ For wave function optimization, CP2K offers both traditional diagonalization schemes and the orbital transformation (OT) method. The OT approach can significantly reduce the computational cost for larger systems while ensuring robust convergence.^38,60,61^

The DFT and GPW formalisms implemented in CP2K provide the foundation for the study of excited-state properties and dynamics. A wide range of advanced excited-state methods have been developed based on DFT within CP2K, which will be discussed in more detail below.

### Linear-Response Time-Dependent Density Functional Perturbation Theory

Linear-response (LR) TD-DFPT in the Sternheimer formalism is particularly useful for calculating excited-state-specific properties, although in a perturbative manner, in contrast with ΔSCF. For instance, TD-DFPT can be employed to calculate reaction paths in photochemical processes or to analyze the molecular orbitals involved in a specific excitation, providing valuable chemical insight.^62^ For such applications, a highly efficient implementation is essential. In this context, we discuss the computational methodologies available for TD-DFPT in CP2K. These include the GPW method for calculating two-electron integrals using either Gaussian basis sets or plane waves,^48^ the computational acceleration of Fock exchange integral calculations,^58,63^ the use of semiempirical kernels in the simplified Tamm–Dancoff approximation,^64^ and the GAPW method.^20^

#### Formalism

In CP2K, excited states are computed using a formulation of TDDFT. While ground state DFT focuses on the total energy as described by eq 1, TDDFT extends this formalism to describe excited states and electronic transitions. In TDDFT, excited states are treated as excitations from the ground state, with excitation energies obtained by solving the Casida equations, also referred to as random-phase approximation (RPA) TDDFT or simply the TDDFT equations.^65^

A widely used approximation to the RPA TDDFT equations, which serve as the foundation for CP2K’s implementation, is the Tamm–Dancoff approximation (TDA).^66^ What distinguishes CP2K’s approach is the reformulation of the TDA equations within the Sternheimer formalism,^67^ eliminating the need for unoccupied molecular orbitals (MOs). This results in an efficient method for large-scale systems, referred to as the TD-DFPT method. The TD-DFPT equation is given by^19^

5 $$\underset{\nu }{\sum }\overset{\mathrm{occ}}{\underset{j}{\sum }}\left(F_{\mu \nu \sigma }\delta_{ij}-S_{\mu \nu }F_{ij\sigma }\right)X_{\nu j\sigma }+\underset{\tau }{\sum }Q_{\mu \tau \sigma }K_{\tau i^{\sigma }}\left[X\right]=\underset{\nu }{\sum }\omega S_{\mu \nu }X_{\nu i\sigma }$$

In eq 5 we find the Kohn–Sham matrix **F**, the overlap matrix **S**, the excitation vectors **X**, and the corresponding excitation energies ω. The operator **K**[**X**] is defined as

6 $$K_{\tau i^{\sigma }}\left[X\right]=\overset{\left\{\alpha ,\beta \right\}}{\underset{\sigma^{\prime }}{\sum }}\underset{\nu }{\sum }\overset{\mathrm{occ}}{\underset{j}{\sum }}\left[\left(\tau i^{\sigma }|\nu j^{\sigma \prime }\right)-a_{\mathrm{EX}}\delta_{\sigma \sigma \prime }\left(\tau \nu |i^{\sigma }j^{\sigma }\right)+\left(\tau i^{\sigma }|f_{\mathrm{xc}}^{\sigma \sigma \prime }|\nu j^{\sigma \prime }\right)\right]X_{\nu j\sigma \prime }$$

where the contribution of exact exchange is controlled by the coefficient *a*_EX_. The two-electron integrals, (*τi* ^σ^|*νj* ^σ′^), are expressed in Mulliken notation, where Greek letters ν, τ refer to atomic orbitals (AOs), *i*, *j* denote indices for occupied (“occ”) MOs, and σ is used to indicate spin-up (α) or spin-down (β). The second functional derivative of the exchange–correlation functional is represented by *f*_xc_^σσ′^. The unoccupied MO space is introduced via the projector **Q**_σ_ onto the unoccupied space, which is defined as

7 $$Q_{\mu \nu \sigma }=\delta_{\mu \nu }-\overset{\mathrm{occ}}{\underset{i}{\sum }}\underset{\tau }{\sum }S_{\mu \tau }C_{\tau i\sigma }C_{\nu i\sigma }$$

The excitation vectors must satisfy the following normalization condition:

8 $$\overset{\left\{\alpha ,\beta \right\}}{\underset{\sigma }{\sum }}\overset{\mathrm{occ}}{\underset{i}{\sum }}\underset{\mu ,\nu }{\sum }X_{\mu i\sigma }S_{\mu \nu }X_{\nu i\sigma }=1$$

In CP2K, analytic derivatives of the excitation energy can be computed using the Z-vector method within the Sternheimer formalism.^19^ The first step involves calculating the response vector **R**. The next step is solving the Z-vector linear equation system, and finally, computing the desired derivative. The Z-vector equation system is given by

9 $$\overset{\mathrm{occ}}{\underset{j}{\sum }}\underset{\nu }{\sum }Z_{\nu j\sigma }\left(F_{\mu \nu \sigma }\delta_{ji}-S_{\mu \nu }F_{ji\sigma }\right)+\underset{\nu }{\sum }Q_{\mu \nu \sigma }H_{\nu i\sigma }\left[Z\right]=-\underset{\nu }{\sum }Q_{\mu \nu \sigma }R_{\nu i\sigma }$$

As can be observed, the Z-vector equation (eq 9) closely resembles the TD-DFPT equation (eq 5), with the primary difference being the introduction of the operator **H**[**Z**], defined as

10 $$H_{\nu i^{\sigma }}\left[Z\right]=2\overset{\left\{\alpha ,\beta \right\}}{\underset{\sigma^{\prime }}{\sum }}\underset{\mu }{\sum }\overset{\mathrm{occ}}{\underset{j}{\sum }}\left[\left(\nu i^{\sigma }|\mu j^{\sigma \prime }\right)-\frac{a_{\mathrm{EX}}\delta_{\sigma \sigma \prime }}{2}\left[\left(\nu \mu |i^{\sigma }j^{\sigma \prime }\right)+\left(\nu j^{\sigma \prime }|i^{\sigma }\mu \right)\right]+\left(\nu i^{\sigma }|f_{\mathrm{xc}}^{\sigma \sigma \prime }|\mu j^{\sigma \prime }\right)\right]Z_{\mu j\sigma \prime }$$

The response vector **R** can be found in ref;^19^ here we focus on the computationally relevant aspects, particularly the calculation of **K**[**X**]. The operator **H**[**Z**] is not discussed due to its similarity to **K**[**X**].

In CP2K, the operator **K**[**X**] is computed within the framework of the GPW^48^ method; it allows the electronic density to be represented either in real space using Gaussian-type orbitals or in reciprocal space with plane waves, with the two representations mapped through a Fourier transformation (see eq 4). Consequently, Coulomb and Fock exchange terms are evaluated in reciprocal space, while the exchange–correlation term is handled via numerical integration in real space. This approach facilitates the inclusion of PBC for extended periodic systems, where long-range Coulomb forces are treated using Ewald summation techniques to ensure convergence over large distances.^68^

The calculation of the Fock exchange term in eq 6 can become a significant computational bottleneck, particularly for large systems. This challenge can be mitigated through the ADMM,^58^ which approximates the Fock exchange term in a smaller basis set. The intrinsic error of this method is alleviated with a correction term based on a local functional. Typical deviations in ADMM results are approximately 0.2 pm for bond lengths of optimized geometries of excited states and 0.02 eV for excitation energies when using triple-ζ basis sets.^19^ Although the formal computational scaling is of fourth order, standard screening techniques can reduce this scaling to below cubic. Moreover, the scaling is with respect to the size of the auxiliary basis set, which is smaller than the primary basis set. As a result, the observed computation times for the Fock exchange term in the **K**[**X**] operator are comparable to those for the exchange–correlation term.^63^

For large systems, the calculation of the **K**[**X**] operator can become computationally prohibitive. In such cases, it can be approximated using semiempirical operators, with one operator handling the Coulomb repulsion and another addressing the Fock exchange terms. These operators can be further adapted to account for PBC. This approach, known as the simplified Tamm–Dancoff approximation (sTDA),^64^ is expected to provide speedups of up to 2 orders of magnitude in the computation of **K**[**X**] while maintaining accuracy comparable to that of the regular TDA.^19,64^

More recently, the GAPW method was implemented for excited states’ analytic derivatives.^20^ One of the key advantages of GAPW over the GPW method is the use of a coarser grid. Despite this, the reduction in accuracy is minimal, with an energy error of around 10^–3^ eV and a force error of 10^–4^ atomic units.^20^ This lower plane wave cutoff proves particularly beneficial for large systems, where the computational efficiency can be improved substantially.

In another recent work^21^ (discussed in Nonadiabatic Molecular Dynamics), the TD-DFPT implementation in CP2K was tailored with a modified version of the Zagreb surface hopping code to perform surface-hopping calculations on potential energy surfaces of TD-DFPT excited states. This state-of-the-art approach, based on a Python script to interface both programs, enabled the calculation of the photodeactivation of *o*- and *p*-nitrophenol species. In this surface-hopping scheme, the probability of hopping to another potential energy surface of the same multiplicity was calculated by the Landau–Zener approach; whereas, the probability of hopping between states of different multiplicities was calculated by the inclusion of spin–orbit coupling between the states.

Building upon these, ongoing developments in the Luber group focus on extending the TD-DFPT framework in CP2K to enable spin-flip excitations originating from a high-spin open-shell reference. This implementation supports collinear and noncollinear exchange–correlation kernels, offering flexibility for different electronic structure scenarios. In parallel, a separate effort is focused on enabling mixed-reference spin-flip excitations,^69,70^ allowing access to spin-pure singlet and triplet excited states via spin-flip transitions.

#### Computational Strategies for Accurate and Efficient TD-DFPT Calculations

When performing DFT and TD-DFPT calculations using the GPW method in CP2K software, several computational aspects need to be carefully considered to achieve accurate results while maintaining efficiency. Two critical factors are the selection of an appropriate grid for the auxiliary basis for the electronic density and the efficient computation of ERIs, particularly for gradient calculations.

The GPW method employs an auxiliary basis of plane waves to represent the electronic density, which allows for efficient treatment of the Hartree terms using FFT. This approach enables the efficient computation and contraction of Coulomb integrals in reciprocal space by solving the Poisson equation.^48^ However, the accuracy of this method depends on the resolution of the real-space grid used for the auxiliary basis.

To achieve the desired accuracy in CP2K calculations, it is essential to converge both the CUTOFF and REL_CUTOFF parameters, which are central to the multigrid scheme used in the GPW method. This scheme employs a hierarchy of real-space grids with different resolutions to efficiently represent Gaussian basis functions. Broad Gaussians are projected onto coarser grids, while narrow ones require finer grids; the total electron density is always evaluated on the finest grid. This hierarchical grid structure reduces computational cost by avoiding the direct evaluation of Gaussian integrals on a single, fine grid, which would be prohibitively expensive for large systems.^48,49,51^ Given this multigrid framework, the CUTOFF parameter defines the PW cutoff for the finest level of the multigrid, directly affecting the accuracy and efficiency of the calculation, while REL_CUTOFF determines how Gaussian products are mapped onto different grid levels based on their spatial extent. A systematic approach to identifying the optimal values involves performing a series of single-point energy calculations with increasing cutoff values^71^ until the total energy stabilizes, indicating that suitable cutoff values have been found.

While the GPW method is highly efficient for many DFT calculations, the treatment of exact exchange in hybrid functionals presents a computational challenge. In these cases, the exchange integrals must be computed in real space, which can become a significant bottleneck in the calculation. The computational cost of these integrals scales as $O\left(N^{4}\right)$ , where *N* is related to the number of basis functions. For gradient calculations, this cost is further increased due to the need to compute derivatives with respect to atomic positions. Specifically, for each two-electron integral, derivatives must be taken with respect to the *x*, *y*, and *z* coordinates of each of the four atoms involved in the integral (*μν*|*λσ*). This results in an additional factor, leading to an overall scaling of approximately 3 × 4 × $O\left(N^{4}\right)$ for the gradient calculations.

To address this computational challenge, CP2K employs several strategies to accelerate the calculation of ERIs. One effective approach is to store the ERIs in memory and reuse them when needed, rather than recalculating them on-the-fly for each iteration. This is particularly beneficial for TD-DFPT gradient calculations, where the calculation of derivative ERIs happens several times due to the symmetry requirements of the supplied matrices. By setting appropriate keywords in the input file, such as MAX_MEMORY and TREAT_FORCES_IN_CORE, we can control the storage and reuse of ERIs, potentially achieving significant speedups without loss of accuracy.

Applications of TD-DFPT for nonadiabatic molecular dynamics simulations will be discussed in Nonadiabatic Molecular Dynamics.

## Results and Discussion

This section is structured into four main parts, each dedicated to a distinct methodological approach with a focus on work done in the Luber group: 1. Delta Self-Consistent Field, 2. Nonadiabatic Molecular Dynamics, 3. Density Functional Perturbation Theory, and 4. Real-Time Time-Dependent Density Functional Theory. Each part includes theoretical background, implementation details, representative applications, and a concluding summary.

### 1 Delta Self-Consistent Field

The essential part of any excited-state dynamics is the excited electronic state and its properties. Obtaining them is more challenging than the ground electronic state properties, as the excited electronic states are higher eigensolutions of the electronic Hamiltonian. Despite their accuracy, wave function-based methods are usually not the choice for large and/or periodic systems due to their high computational cost. The delta self-consistent field (ΔSCF) method can optimize an excited electronic state of interest at the DFT level of theory with a computational cost comparable to the ground electronic state DFT SCF optimization. It is an attractive choice for obtaining a specific excited electronic state and its properties, of interest in systems within a dense manifold of excited electronic states, for instance, due to a large number of spectator electronic states present on the solute molecules or the surface, which do not interact with the chromophore. Furthermore, being a variational method, ΔSCF avoids the linear response regime and does not require time adiabaticity approximation like the TDDFT-based techniques. This makes it more suitable for the description of Rydberg and charge transfer excited electronic states,^72–76^ as these are typically underestimated with TDDFT methods.^77–80^ The ΔSCF method, similar to the complete active space methods, relaxes the molecular orbitals of each excited electronic state, and can account for double excitation excited electronic states that the linear-response TDDFT methods cannot.^75,81,82^

#### 1.1 Formalism

The DFT-based ΔSCF method represents a way of obtaining excited electronic states variationally at the DFT level.^83–88^ For example, in a closed-shell system with *N*_e_ electrons, where *N*_e_ is an even number, whose ground electronic state (S0) configuration |Ψ^S0^⟩ can be represented well with a single Slater determinant,

11 $$\left|\Psi^{S0}\right\rangle =\frac{1}{\sqrt{N_{e}!}}\det\left(\varphi_{1,\alpha }^{S0}\alpha ,\varphi_{1,\beta }^{S0}\beta ,...,\varphi_{o,\alpha }^{S0}\alpha ,\varphi_{o,\beta }^{S0}\beta ,...,\varphi_{N_{e}/2,\alpha }^{S0}\alpha ,\varphi_{N_{e}/2,\beta }^{S0}\beta \right)$$

an electron can be promoted from the highest-energy occupied molecular orbital (HOMO) of the β spin channel into the lowest-energy occupied molecular orbital (LUMO) of the α channel, giving the system’s triplet electronic state (T1) with *M*_*s*_ = +1:

12 $$\left|\Psi^{T1\left(+1\right)}\right\rangle \approx \frac{1}{\sqrt{N_{e}!}}\det\left(\varphi_{1,\alpha }^{T1}\alpha ,\varphi_{1,\beta }^{T1}\beta ,...,\varphi_{o,\alpha }^{T1}\alpha ,\varphi_{o,\beta }^{T1}\beta ,...,\varphi_{N_{e}/2,\alpha }^{T1}\alpha ,\underset{\_}{\varphi_{N_{e}/2+1,\alpha }^{T1}\alpha \;}\right)$$

The underline emphasizes the changed Kohn–Sham (KS) MO, and the other degenerate triplet state with *M*_*s*_ = – 1 can be constructed similarly. In the above expressions, the φ stands for the KS spatial MOs, while the **α** and **β** are the spin-up and spin-down functions, respectively. Indices of occupied KS MOs (*j* in subscript) range from 1 to *N*_e_/2, or *N*_e_/2 + 1, and follow a usual convention of ordering the MOs by their corresponding eigenvalues, with 1 associated with the lowest eigenvalue. In addition, a spin index σ is also associated with every MO as they can differ between the two spin channels. The following relation gives the electronic density for each spin channel ρ_σ_^*i*^(**r**)­

13 $$\rho_{\sigma }^{i}\left(r\right)=\underset{j}{\sum }n_{j\sigma }^{i}{\left|\varphi_{j\sigma }^{i}\left(r\right)\right|}^{2}$$

where *n* is the occupation number associated a given MO. For the S0 state, all KS MOs within the range 1 to *N*_e_/2 have occupation 1, while for the T1­(+1) state, one MO with index *N*_e_/2 + 1, which in the S0 state was an unoccupied virtual MO (the LUMO), gains occupation 1, while the *N*_e_/2 MO (the HOMO in the S0 state) of the other spin channel has now occupation 0. The DFT energy expression

14 $$E^{i}=\overset{\left\{\alpha ,\beta \right\}}{\underset{\sigma }{\sum }}\int \left\{-\frac{1}{2}\underset{j}{\sum }n_{j\sigma }^{i}\varphi_{j\sigma }^{i}\left(r\right)\nabla^{2}\varphi_{j\sigma }^{i}\left(r\right)+(w\left(r\right)+\frac{1}{2}\int \frac{\rho^{i}\left(r^{\prime }\right)}{\left|r-r^{\prime }\right|}dr^{\prime }+v_{\mathrm{xc}}^{\sigma }\left[\rho_{\alpha }^{i},\rho_{\beta }^{i}\right]\left(r\right))\rho_{\sigma }^{i}\left(r\right)\right\}dr$$

gives the energies of states in eqs 11 and 12.^89^ The individual terms on the right-hand-side of eq 14 are the kinetic energy (∇^2^ = ∂^2^/∂*x* ^2^ + ∂^2^/∂*y* ^2^ + ∂^2^/∂*z* ^2^), the external potential *w*(**r**), the Coulomb or Hartree potential (1/2 ∫d**r**′ ρ­(**r**′)/|**r** – **r**′|), and the exchange–correlation (xc) functional, respectively. The brackets in the xc term designate its functional dependence on the electronic spin densities, where the higher derivatives of the electronic densities with respect to **r** (see eq 4) are omitted to simplify eq 14. Likewise, the electrostatic nuclear energy contribution omitted from eq 14 for simplicity, as it does not depend on the electronic part, but the total electron energy also includes this term. The total electron density ρ^*i*^(**r**) is ρ_α_^*i*^(**r**) + ρ_β_^*i*^(**r**). The KS MOs are optimized to minimize the DFT energy for configurations in eqs 11 and 12. Note that for each optimized electronic state, the KS MO are unrelated and not mutually orthogonal. For this reason, the superscript *i* is used along each term that depends on the electronic state of interest. The Hohenberg–Kohn (HK) theorem justifies the construction of such excited electronic states.^90^ The triplet electronic state is not directly accessible by photoabsorption from the S0 state, but the singlet excited electronic state S*i* is, whose configuration is

15 $$\left|\Psi^{\mathrm{Si}}\right\rangle \approx \frac{1}{\sqrt{2N_{e}!}}\left\{\det\left(\varphi_{1}^{\mathrm{Si}}\alpha ,\varphi_{1}^{\mathrm{Si}}\beta ,...,\underset{\_}{\varphi_{v}^{\mathrm{Si}}\alpha \;},\varphi_{o}^{\mathrm{Si}}\beta ,...,\varphi_{N_{e}/2}^{\mathrm{Si}}\alpha ,\varphi_{N_{e}/2}^{\mathrm{Si}}\beta \right)+\det\left(\varphi_{1}^{\mathrm{Si}}\alpha ,\varphi_{1}^{\mathrm{Si}}\beta ,...,\varphi_{o}^{\mathrm{Si}}\alpha ,\underset{\_}{\varphi_{v}^{\mathrm{Si}}\beta \;},...,\varphi_{N_{e}/2}^{\mathrm{Si}}\alpha ,\varphi_{N_{e}/2}^{\mathrm{Si}}\beta \right)\right\}$$

One notes several distinctions of the singlet excited state in eq 15 with the triplet excited state (eq 12). First, for the state to be of pure singlet multiplicity (*S*_*z*_ = 0), the KS MOs between the two spin channels must be equal. The spin index can be omitted from the spatial MOs for this reason. Second, the state is composed of two Slater determinants instead of one. Last, the excitation can happen from any initially occupied (*o*) KS MO to any virtual (*v*) KS MO. This terminology follows the MOs’ designations in the ground electronic states as the spatial MOs of the excited electronic states still closely resemble those of the S0 state. While the MOs can be restrained to be equal between the two spin channels via the restricted open-shell KS (ROKS) DFT formulation, the electronic state’s multideterminant construction is not naturally associated with DFT. It should be noted that the above singlet excited state is represented by just one configuration, meaning just one contribution for the electron promotion from an occupied to a virtual MO. In reality, all electronic states, including the S0 state, can be represented as infinite expansions into configurations, where usually just a few dominate.

While every stationary excited electron state has a corresponding electronic density, obtaining the excited electronic state properties from the electronic density alone is not straightforward. The ΔSCF method was justified in a series of works by Ayers, Görling, Levy, Nagy, and others.^91–95^ In essence, a mapping between excited-state density and excited electronic state properties is possible within the KS DFT formulation, but the electronic density alone does not represent the complete information on an excited electronic state.^94^ Instead, a specialized xc functional that contains additional information on the excited electronic state is required.^96^ Finally, the connection between the full interaction electronic wave function and its noninteracting KS counterpart is assured via the adiabatic connection.^91^ While theory assures that such xc functionals exist, in practice, no such xc functional is readily available for immediate application. Nonetheless, a crude but effective adiabatic approximation of directly using the ground state xc functional for excited-state optimizations is generally made.

A typical DFT excited-state optimization procedure is to employ the SCF algorithm. As the KS MOs are ordered by energy, MO root-flipping can occur and the electronic density can lose track of its constituting MOs, mismatching the target electron density and ending in the variational collapse. Thus, the initial singlet excited state usually optimizes back to the S0 state. Generally, the excited electronic states are stationary points in the space spanned by the MOs, with several negative eigenvalues if their corresponding MO Hessians are diagonalized, which makes the excited-state optimizations challenging. A tracking procedure for occupation numbers or MOs, based on the maximum overlap method (MOM),^82,97,98^ can be utilized to maintain the electronic density in line with the target excited electronic state during the regular SCF optimization. The alternative is directly optimizing the MOs by quasi-Newton-based methods, which can directly converge to stationary points of interest.^99,100^

The CP2K program package was adapted with several techniques that enable variational construction of excited electronic states via the ΔSCF method. The following sections explain how the aforementioned challenges of the ΔSCF method were addressed.

#### 1.2 Implementation Details in CP2K

##### Spin Purification

The triplet excited electronic state with *M*_*s*_ = 0 is analogous to the singlet excited state in eq 15,

16 $$\left|\Psi^{Ti\left(0\right)}\right\rangle \approx \frac{1}{\sqrt{2N_{e}!}}\left\{\det\left(\varphi_{1}^{Ti}\alpha ,\varphi_{1}^{Ti}\beta ,...,\underset{\_}{\varphi_{v}^{Ti}\alpha \;},\varphi_{o}^{Ti}\beta ,...,\varphi_{N_{e}/2}^{Ti}\alpha ,\varphi_{N_{e}/2}^{Ti}\beta \right)-\det\left(\varphi_{1}^{Ti}\alpha ,\varphi_{1}^{Ti}\beta ,...,\varphi_{o}^{Ti}\alpha ,\underset{\_}{\varphi_{v}^{Ti}\beta \;},...,\varphi_{N_{e}/2}^{Ti}\alpha ,\varphi_{N_{e}/2}^{Ti}\beta \right)\right\}$$

and, unlike the triplet *M*_*s*_ = ±1 states (eq 12), is not directly available via the DFT optimization. In case the MOs of the singlet (eq 15) and triplet *M*_*s*_ = 0 (eq 16) states are equal, their linear combination

17 $$\left|\Psi^{Si}\right\rangle +\left|\Psi^{Ti\left(0\right)}\right\rangle =\frac{2}{\sqrt{2N_{e}!}}\det\left(\varphi_{1}^{i}\alpha ,\varphi_{1}^{i}\beta ,...,\underset{\_}{\varphi_{v}^{i}\alpha \;},\varphi_{o}^{i}\beta ,...,\varphi_{N_{e}/2}^{i}\alpha ,\varphi_{N_{e}/2}^{i}\beta \right)$$

yields a single Slater determinant with the electron promoted from an occupied (*o*) to a virtual (*v*) MO within the same spin channel. Since the energy of the triplet *M*_*s*_ = 0 state (eq 16) is degenerate with the triplet *M*_*s*_ = ±1 state, which can be constructed as in eq 12, the energy of the above linear combination becomes *E* ^S*i*^ + *E* ^T*i*(±1)^ = 2*E* ^(S+T)*i*^. From it, the singlet excited state energy is

18 $$E^{Si}=2E^{\left(S+T\right)i}-E^{Ti\left(\pm 1\right)}$$

where each of the right-hand-side energies is obtained from a single Slater determinant. With the same set of KS MOs the states S*i*, T*i*(0), T*i*(±1), and (S + T)*i* all have the same total electronic density but mutually differ in the spin densities, with the first two being equal and different from the rest. Equation 18 illustrates the spin purification (SP) approach for constructing singlet excited electronic states.^83^ It was generalized to several states of other multiplicities.^101,102^ Despite that the derivation requires the same KS MOs between the two Slater determinants (eqs 12 and 17), in various examples^73,79,83,99,102–105^ the unrestricted KS (UKS) formulation was used to independently optimize each energy contribution from which an approximation of the singlet excitation is made.^106^ Needless to say, no such independent optimization of constituting electronic densities can yield the singlet excited state electronic density, and no other observables can be accurately accessed from the UKS ΔSCF. To obtain the singlet excited state energy from eq 18, the same energy construction must be used in the optimization of the ROKS MOs. This involves employing two electronic densities—corresponding to the triplet (eq 12) and the singlet–triplet superposition (eq 17)—within the KS and energy expression (eq 14).^22,106^ With the optimized KS MOs, the singlet excited state electron density is easily constructed (eq 19), and other observables can be obtained (see later). This procedure was implemented in CP2K.^22^

##### Direct Singlet Excited States

The singlet excited electronic state can also be directly constructed within the R­(O)­KS scheme.^23,61,107–109^ The corresponding electron spin density for the singlet excited electronic state given by eq 15 is

19 $$\rho_{\sigma }^{Si}\left(r\right)={\left|\varphi_{1}^{Si}\left(r\right)\right|}^{2}+...+\frac{1}{2}{\left|\varphi_{o}^{Si}\left(r\right)\right|}^{2}+\frac{1}{2}{\left|\varphi_{v}^{Si}\left(r\right)\right|}^{2}+...+{\left|\varphi_{N_{e}/2}^{Si}\left(r\right)\right|}^{2}$$

which contains half-occupied *o* and *v* KS MOs. In other words, half an electron is promoted from an initially fully occupied MO to an empty virtual MO within each spin channel to get an excited singlet electronic state. The energy of this singlet excited electronic state is obtained by directly including the above electron density into the energy expression in eq 14, which is also minimized during the standard KS MO optimization.^23,24,61,89,108,109^ One notices that the triplet *M*_*s*_ = 0 state has the same spin density (eq 19) as the singlet excited state. However, its corresponding energy obtained via eq 14 would not give the same value as for triplet *M*_*s*_ = ±1 states because the xc functional assigns lower energy multiplicity values to states with *M*_*s*_ = ±(*M*_*s*,max_ – 1).^110^ In the case of triplet *M*_*s*_ = 0 states, for which *M*_*s*,max_ = 1, this would correspond to the singlet state energy, as the two spin densities are equal.

As aforementioned, this and the previous SP approach, enable only a description of the single-configuration excited electronic states. It is possible to generalize the electron density expression to include multiple single-configuration excitation^111^ contributions as

20 $$\left|\Psi^{Si}\right\rangle \approx \frac{1}{\sqrt{2}}\underset{o\in X\left(o\right),v\in X\left(v\right)}{\sum }C_{ov}^{Si}\left(\left|\Phi_{o\alpha \to v\alpha }^{Si}\right\rangle +\left|\Phi_{o\beta \to v\beta }^{Si}\right\rangle \right)$$

where |Φ_*oα*→*vα*_^S*i*^⟩ and |Φ_*oβ*→*vβ*_^S*i*^⟩ stand for the determinants explicitly written on the right-hand-side of eq 15. The factor $1/\sqrt{N_{e}!}$ is assimilated into the right-hand side ket terms. X is the set {..., *o* → *v*, ...} of all included single excitation types to approximate the excited electronic state, where X(o) and X(v) designate the initially occupied and its substituting virtual MO, respectively. While this expands the electron density with additional MOs, the corresponding energy and other properties are still single-configuration type as the underlying DFT does not support multiconfiguration description. The occupation numbers for such electron density are

21 $$n_{j\sigma }=\{\begin{array}{ll} 1, & j\leq N_{e}/2\wedge j\notin X\left(o\right)\\ 1-\frac{1}{2}\underset{v\in X\left(v\right)}{\sum }{\left|C_{jv}^{Si}\right|}^{2}, & j\in X\left(o\right)\\ \frac{1}{2}\underset{o\in X\left(o\right)}{\sum }{\left|C_{oj}^{Si}\right|}^{2}, & j\in X\left(v\right)\\ 0, & j>N_{e}/2\wedge j\notin X\left(v\right) \end{array}$$

The coefficients *C*_*ov*_^S*i*^ are fixed here, obtained from another method, e.g., TD-DFPT, and while their phases (signs) do not influence the electronic density, they are important in determining other observables (see Observables).^111^

##### ΔSCF Convergence

The excited electronic state energy is obtained by optimizing the DFT energy (eq 14) with two restraints: (1) the KS MOs must remain orthonormal, i.e., MOs must satisfy the relation ∫φ_*j*_^*i*^(**r**) φ_*k*_^*i*^(**r**) d**r** = δ_*jk*_, where δ_*jk*_ is the Kronecker delta, and (2) the total and spin electronic densities should match the corresponding values of the target excited electronic state that one wants to obtain. Given that the excited electronic states are optimized separately, there is no restriction between them being mutually orthogonal, and thus their corresponding KS MOs are not mutually orthonormal. The MO orthonormality within the electronic state is assured by inserting this requirement into the energy Lagrangian, which leads to the KS equations

22 $$\left\{-\frac{1}{2}\nabla^{2}+v_{\mathrm{xc}}^{\sigma }\left[\rho_{\alpha }^{i},\rho_{\beta }^{i}\right]\left(r\right)+w\left(r\right)+\int \frac{\rho^{i}\left(r^{\prime }\right)}{\left|r-r^{\prime }\right|}dr^{\prime }\right\}\varphi_{j}^{i}\left(r\right)=\varepsilon_{j}^{i}\varphi_{j}^{i}\left(r\right)$$

with the familiar terms in curly brackets on the left-hand side (see eq 14), and ε_*j*_^*i*^ is the KS MO corresponding eigenvalue. The procedure requires some knowledge of the nature of the target excited electronic state, which is to be constructed with the ΔSCF method. This can be obtained from a high-level wave function method, TD-DFPT, or even from an educated guess.

The KS MOs have to be solved iteratively by the SCF procedure. Within ΔSCF, it is paramount that the electron density in every SCF step corresponds to the target electronic density of the excited electronic state. One starts with the idea of the target state electronic density. The reference electronic density, to compare the target electronic density, can be constructed by carefully assigning proper occupation numbers to the ground electronic state MOs, and can be used as the starting guess electronic density in the SCF procedure. As the SCF diagonalization algorithm orders the MOs by their corresponding eigenvalues, it easily happens that the new MOs are ordered differently than in the previous SCF step. This causes a mismatch between MOs and the initially assigned occupation numbers, so the electron density deviates from the target one. In these cases, it becomes necessary to track the MOs or their associated occupation numbers throughout the SCF procedure to maintain the target electronic density and achieve convergence. The MOM is usually sufficient for maintaining the correct association of occupation numbers with the corresponding MOs in the SP ΔSCF schemes, and the reader is reminded that with the original MOM, the occupation numbers are reordered.^82,97,98^ However, the MOM method is insufficient for keeping the correct electron density if fractional occupation numbers are used.

For this purpose, we implemented two procedures, based on the overlap comparison, into CP2K (Figure 1).^111^ In the adapted initial MOM (AIMOM) procedure, the reference MOs are weighted with the occupation numbers and overlapped with the current MOs.^111^ If the singlet excited state is optimized directly two overlap values will be close to 1/2. Their positions are marked, and for both the occupation number of 1/2 is assigned. The list with occupation numbers is reordered according to the MOs that needed to be tracked (see Figure 1). The procedure is repeated in each SCF step. In the other procedure, named Switcher, the current MOs are overlapped with a few selected reference MOs.^111^ This evaluates the similarity between the current and reference MOs. If the current MO is similar and ordered exactly as the reference MO, then the indices *j* and *k* coincide, otherwise, the Switcher reorders the current MO to match the ordering of the reference MO (see Figure 1). Unlike the AIMOM procedure, the Switcher enables simultaneous and precise tracking of multiple MOs when the electronic density is described by more than a pair of fractional occupation numbers (eq 21). A comparison between the two procedures and their advantages is shown later.

**Figure 1:**
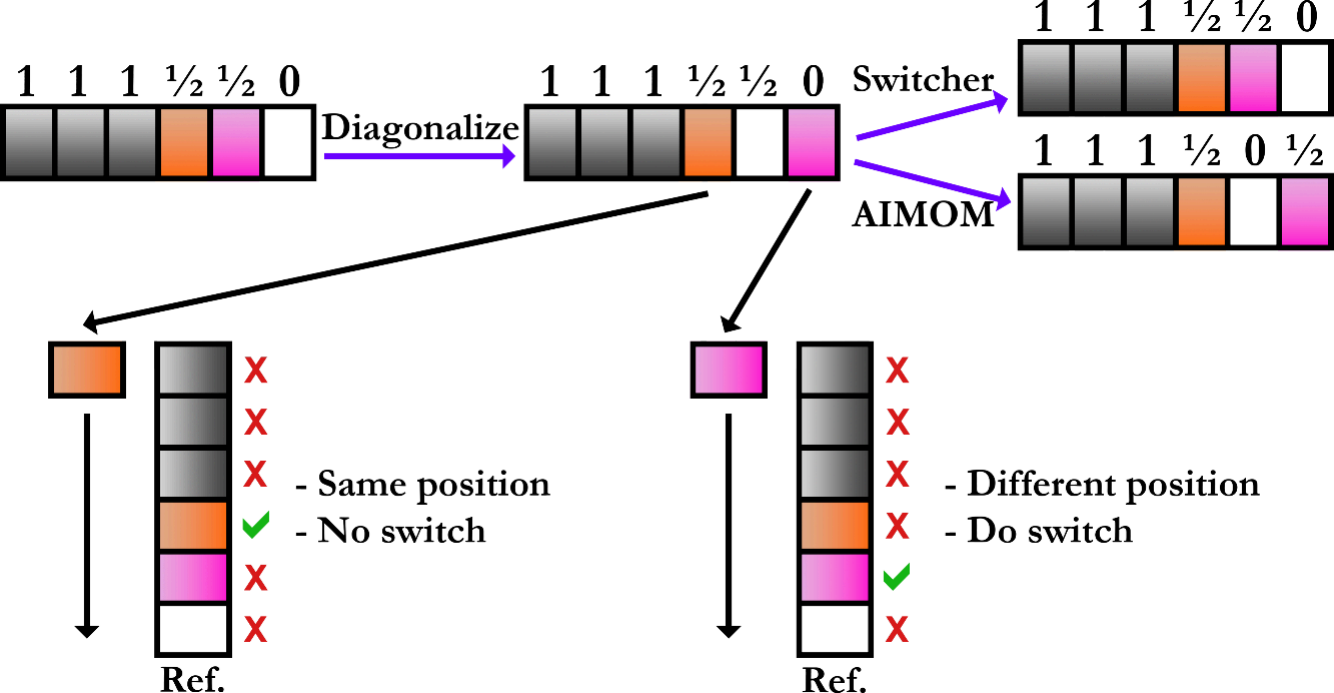
Graphical representations of the AIMOM and Switcher algorithms in the ΔSCF SCF convergence. The bins represent the MOs, where the colored ones are selected for tracking with the Switcher algorithm. The numbers above the bins are the occupation numbers. Adapted from ref 111. Copyright 2025 American Chemical Society.

Sometimes during the SCF optimization, the virtual space MOs interfere too often with the selected MOs. This can be mitigated by applying the level shift option, which systematically raises the energy of all virtual MOs. An advanced-level shift procedure called STEP by Carter-Fenk and Herbert^112^ has also been included in CP2K.^61^

##### Orbital Transformation in ΔSCF

An alternative to optimization by direct diagonalization of the KS eigenvalue equation (eq 22) is the optimization of the MOs.^61,100,113,114^ In the AO basis of size *M*, MOs are transformed, or rotated, according to **C**_*j*+1_^*i*^ = **C**_*j*_^*i*^ exp­(**A**(**X**)) until the total energy reaches a stationary point, where the gradient of the energy with respect to the MO coefficients, but also with respect to **X**, vanishes, i.e., ∂*E* ^*i*^/∂**C** ^*i*^ ≡ ∂*E* ^*i*^/∂**X** = 0. The matrices **C**_*j*+1_^*i*^ and **C**_*j*_^*i*^ contain the MO coefficients at two consecutive iteration steps, *j* + 1 and *j*, respectively. Together with the anti-Hermitian matrix **A** they are of *M* × *M* dimensions. Matrix **X** represents the free variables that rotate the MOs, and with it, they tune the energy. They are linearly constrained to remain orthogonal to the starting occupied MOs, **X** ^†^ **SC**_O_ = **0**,^60^ where the **C**_O_ are the *N*_O_ occupied MOs in **C**_*j*_^*i*^. The matrices **X** and **C**_O_ are of dimension *M* × *N*_O_, where *N*_O_ stands for the number of occupied MOs. For the ground electronic state, *N*_O_ is *N*_e_/2, while for the singlet and triplet excited electronic states *N*_O_ is *N*_e_/2 + 1. In the R­(O)­KS ground electronic state, the diagonal subblock **A**_OO_ of dimension *N*_e_/2 × *N*_e_/2 which rotates the occupied MOs, would also vanish as the energy is invariant to the linear combination of occupied MOs. However, for electronic states with fractional occupation numbers, this invariance breaks and additional terms need to be considered. For details, see the reference.^61^

#### 1.3 Applications

The previously listed ΔSCF procedures implemented into CP2K were applied for different cases.^23,24,61,88,89,111,115^ Several applications are shortly presented here.

##### Observables

While the expectation values of the operators for each ΔSCF constructed excited electronic state are easily obtained by applying the ground electronic state DFT procedures, transition probabilities of an operator between two different electronic states are affected by the fact that the ΔSCF electronic states are not mutually orthogonal. To mitigate this issue and avoid cumbersome expressions which would contain the overlap elements between MOs of two independently optimized electronic states, an expansion procedure based on configuration interaction singles (CIS) was developed.^89^ Based on it, the approximated state wave functions are expanded into a linear combination of singly excited Slater determinants constructed from ground electronic state KS MOs. For example, the singlet excited electronic state (eq 15) takes a form similar to eq 20:

23 $$\left|{\mathrm{\Psi ̃}}^{Si}\right\rangle \approx \frac{1}{\sqrt{2}}\overset{N_{e}/2}{\underset{o=1}{\sum }}\overset{M}{\underset{v=N_{e}/2+1}{\sum }}{\mathrm{C̃}}_{ov}^{Si}\left(\left|\Phi_{o\alpha \to v\alpha }^{S0}\right\rangle +\left|\Phi_{o\beta \to v\beta }^{S0}\right\rangle \right)$$

where the right-hand side Slater determinants are made of S0 state MOs, and the summation now goes over all S0 state’s occupied and unoccupied MOs. The tilde symbol (Ψ̃) distinguishes the CIS expansions from the original wave functions Ψ. The corresponding coefficients *C̃*_*ov*_ are the projections of the approximated wave functions to the right-hand sides of the CIS expansion (eq 23), and for the singlet excited electronic state they are

24 $${\mathrm{C̃}}_{pw}^{Si}=\frac{1}{\sqrt{2}}\left(\left\langle \Phi_{p\alpha \to w\alpha }^{S0}\right|\Psi^{Si}\rangle +\left\langle \Phi_{p\beta \to w\beta }^{S0}\right|\Psi^{Si}\rangle \right)=\left\langle \Phi_{p\alpha \to w\alpha }^{S0}\right|\Phi_{o\alpha \to v\alpha }^{Si}\rangle +\left\langle \Phi_{p\beta \to w\beta }^{S0}\right|\Phi_{o\alpha \to v\alpha }^{Si}\rangle$$

After calculating all projections, they are normalized so that the CIS expansion satisfies ⟨Ψ̃^*i*^|Ψ̃^*i*^⟩ = 1. The CIS expansion is sufficient enough as it captures >99.9% of the original wave function.^89^ It should be noted that the CIS projection does not make the states completely orthogonal to each other, but because it removes the overlap with the ground electronic state, which is the largest contribution, the ⟨Ψ̃^*i*^|Ψ̃^*j*^⟩, for *i* ≠ *j*, becomes almost negligible. The CIS expansion for triplet excited electronic states are similar in form and can be found in reference,^89^ and the CIS expansion of the excited electronic states approximated multiconfiguration wave functions ([Disp-formula eq20]) can be found in reference.^111^

Now values of operators ⟨Ψ^*i*^|*Ô*|Ψ^*j*^⟩, where *Ô* is some hermitian operator, can be easily determined using the CIS expansion on ΔSCF excited electronic states. For example, the transition dipole moment (TDM) operator between the singlet ground state (eq 11) and singlet excited electronic state (eq 15) is

25 $$\left\langle \Psi^{S0}\right|\mathrm{\mu ̂}\left|\Psi^{Si}\right\rangle \approx \left\langle \Phi^{S0}\right|\mathrm{\mu ̂}\left|{\mathrm{\Psi ̃}}^{Si}\right\rangle =\frac{1}{\sqrt{2}}\underset{pw}{\sum }{\mathrm{C̃}}_{pw}^{Si}\left(\left\langle \Phi^{S0}\right|\mathrm{\mu ̂}\left|\Phi_{p\alpha \to w\alpha }^{S0}\right\rangle +\left\langle \Phi^{S0}\right|\mathrm{\mu ̂}\left|\Phi_{p\beta \to w\beta }^{S0}\right\rangle \right)=\sqrt{2}\underset{pw}{\sum }{\mathrm{C̃}}_{pw}^{Si}\left\langle \varphi_{p}^{S0}\right|\mathrm{\mu ̂}\left|\varphi_{w}^{S0}\right\rangle .$$

The electric dipole moment vector operator **μ̂**, which is either in the length or velocity representation, starts as an *N*_e_-electron operator in the above relation but is reduced to a one-electron operator in the final line of eq 25. The spin–orbit coupling (SOC) electron–nuclear term between the excited singlet and triplet states is

26 $$\left\langle \Psi^{Si}\right|{Ĥ}_{\mathrm{SOC}}\left|\Psi^{Tj\left(0\right)}\right\rangle \approx i\frac{\alpha^{2}}{2}\left(\underset{pww^{\prime }}{\sum }{\mathrm{C̃}}_{pw}^{Si}{\mathrm{C̃}}_{pw\prime }^{Tj}\left\langle \varphi_{w}^{S0}\right|{\mathrm{\xi ̂}}_{z}\left|\varphi_{w\prime }^{S0}\right\rangle -\underset{pp^{\prime }w}{\sum }{\mathrm{C̃}}_{pw}^{Si}{\mathrm{C̃}}_{p\prime w}^{Tj}\left\langle \varphi_{p\prime }^{S0}\right|{\mathrm{\xi ̂}}_{z}\left|\varphi_{p}^{S0}\right\rangle \right)$$

and

27 $$\left\langle \Psi^{Si}\right|{Ĥ}_{\mathrm{SOC}}\left|\Psi^{Tj\left(\pm 1\right)}\right\rangle \approx i\frac{\alpha^{2}}{2^{3/2}}\left(\underset{pww^{\prime }}{\sum }{\mathrm{C̃}}_{pw}^{Si}{\mathrm{C̃}}_{pw\prime }^{Tj}\left\langle \varphi_{w}^{S0}\right|{\mathrm{\xi ̂}}_{x}\pm i{\mathrm{\xi ̂}}_{y}\left|\varphi_{w\prime }^{S0}\right\rangle -\underset{pp^{\prime }w}{\sum }{\mathrm{C̃}}_{pw}^{Si}{\mathrm{C̃}}_{p\prime w}^{Tj}\left\langle \varphi_{p\prime }^{S0}\right|{\mathrm{\xi ̂}}_{x}\pm i{\mathrm{\xi ̂}}_{y}\left|\varphi_{p}^{S0}\right\rangle \right)$$

where three components of the electron–nuclear SOC operator ξ̂ are

28 $${\mathrm{\xi ̂}}_{j}={\left\{\overset{N_{n}}{\underset{n=1}{\sum }}\frac{Z_{n}^{*}}{|r_{n}{|}^{3}}\left({\mathrm{r̂}}_{n}\times \nabla \right)\right\}}_{j},,\mathrm{for}\;j=x,y,z$$

*Z*_n_^*^ stand for the effective nuclear charge while **r**_*n*_ for the distance between *n*-nucleus and electron, whose contributions have to be sum over all *N*_*n*_ nuclei, while α is a fine structure constant, and i an imaginary unit. The expressions in eqs 25–27 are mathematically identical to the linear-response TDDFT corresponding relations obtained by the auxiliary wave functions.^116–124^ The only difference is the origin of the *C̃* terms. For NAMD purposes, the SOC terms (eqs 26 and 27) are assembled into one effective SOC term as^122,125,126^

29 $$\left\langle \Psi^{Si}\right|{Ĥ}_{\mathrm{SOC}}\left|\Psi^{Tj}\right\rangle ={\left(\underset{M_{s}=+1,0,-1}{\sum }{\left|\left\langle \Psi^{Si}\right|{Ĥ}_{\mathrm{SOC}}\left|\Psi^{Tj\left(M_{s}\right)}\right\rangle \right|}^{2}\right)}^{1/2}$$

An example of SOC calculation is presented in Figure 2.^89^

**Figure 2:**
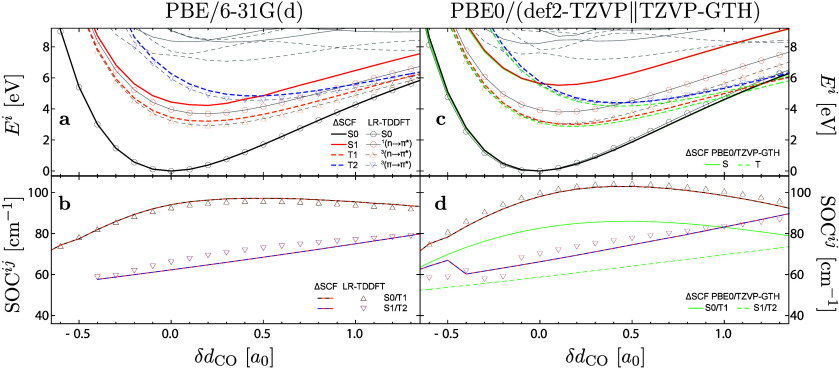
Energy profile of several singlet (full lines) and triplet (dashed lines) electronic states along the relative change of the C=O bond length (δ*d*_CO_, in bohr) in formaldehyde. 0.0 corresponds to the ground-state equilibrium bond length. In plots (a) and (c), black, red, orange, and blue lines indicate S0, S1, T1, and T2 ΔSCF electronic states, respectively. Gray lines indicate adiabatic TD-DFPT electronic states, where the black and red circles trace the singlet ground and ^1^(n → π*) characters, respectively, while orange and blue triangles trace the triplet ^3^(n → π*) and ^3^(π → π*) characters, respectively. Plots (b) and (d) show the one-electron SOC terms between S0 and T1 electronic states (in black/orange) and between S1 and T2 (in red/blue). Full lines indicate ΔSCF, while triangles trace the TD-DFPT values. All values in (a) and (b) were obtained using the PBE^127,220^ xc functional with the 6-31G­(d) basis set. In (c) and (d) the PBE0 ^128^ functional was combined with the all-electron def2-TZVP basis set^129^ or with the TZVP-GTH basis set^59^ used exclusively with the ΔSCF method. Corresponding values computed with the TZVP-GTH basis set are indicated by green lines in (c) and (d) and are paired with the corresponding all-electron counter curves. Adapted from ref 89. Copyright 2022 American Chemical Society.

##### Convergence Benchmarks

The ΔSCF CP2K implementation was extensively benchmarked.^111^ For the first time, the 20 lowest singlet and 20 lowest triplet excited electronic states were computed at the ΔSCF level of theory and compared to TDDFT. These excited electronic states span from dominantly single-configuration up to multiconfiguration characters, over a wide range of molecules, classified into three distinctive types.^111^ The excited states were first obtained with TDDFT and for each excited state, the corresponding occupation numbers were generated using eq 21, as explained in ref 111. In addition to comparing the excitation energies (difference between the excited-state and ground-electronic-state energies at the ΔSCF level), TDMs for each state were examined (their norms and directions separately) as well as the excited electronic state densities. Given that the ΔSCF method generates the excited-state electron density, while the TDDFT immediately gives the density difference, the density difference δρ^*i*^ was also constructed at the ΔSCF level by taking the difference between the excited and ground electronic state densities. This density difference δρ^*i*,ΔSCF^ is affected by the difference between the MOs, since ρ^*i*,ΔSCF^ is derived from the different MOs associated with the corresponding state *i*, while the TDDFT uses only the S0 state MOs. A similarity value η^*i*^ between two density differences is defined as

30 $$\eta^{i}=\exp\left[-\frac{{\left(\underset{j}{\sum }\left|\delta \rho_{j}^{i,\Delta \mathrm{SCF}}-\delta \rho_{j}^{i,\mathrm{TDDFT}}\right|\right)}^{2}}{\underset{j}{\sum }\left|\delta \rho_{j}^{i,\Delta \mathrm{SCF}}\right|\cdot \underset{j}{\sum }\left|\delta \rho_{j}^{i,\mathrm{TDDFT}}\right|}\right]$$

and is given in percentage.^111^ The summation goes over all grid points.

Figure 3 graphically summarizes the comparison between ΔSCF and TDDFT for one class of molecules examined in.^111^ Without any SCF convergence assistance, the average density differences show a larger discrepancy with the TDDFT as some states converged to states different from the target electronic states. Consequentially the similarity of observables between the two methods reduces. The AIMOM and the Switcher algorithms (see ΔSCF Convergence) greatly improve the convergence and the results.

**Figure 3:**
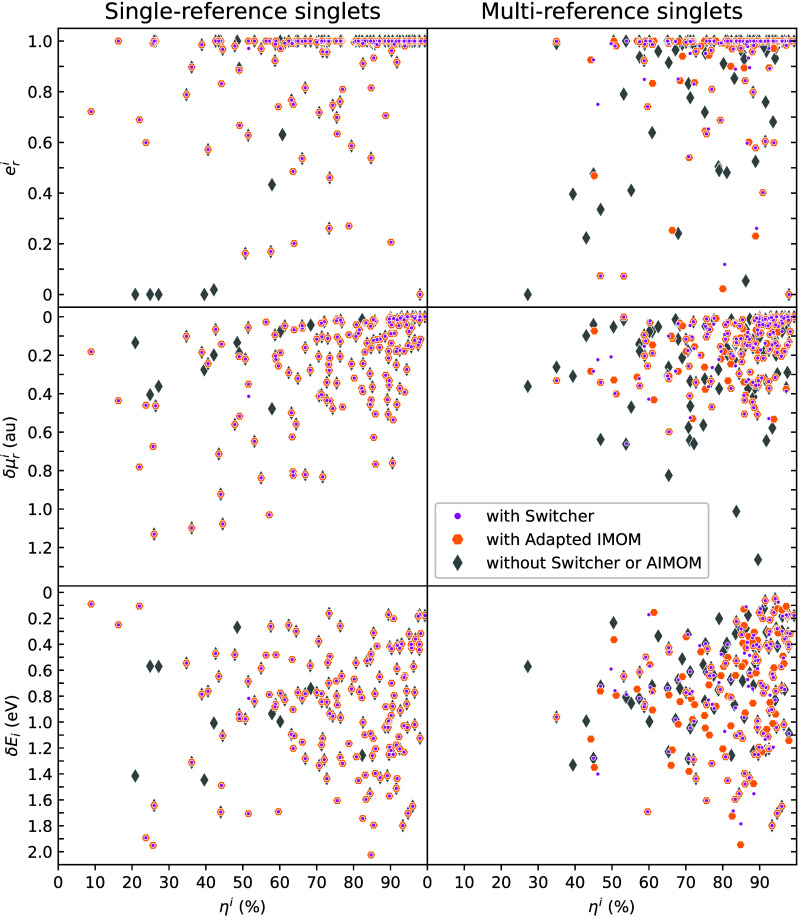
Comparison between ΔSCF and TD-DFPT results obtained for several excited electronic states classified as single- and multiconfiguration. Adapted from ref 111. Copyright 2025 American Chemical Society.

For the OT procedure (see in Orbital Transformation in ΔSCF), benchmark calculations were conducted on a set of aromatic and heteroaromatic molecules, comparing their first two singlet excited L_*a*_ and L_*b*_ states with the ones from TD-DFPT and RI-CC2 values.^61^ In addition, the methodology was applied to obtain the first singlet excited electronic states for solvated ethylene and uracil molecules in periodic boxes containing 27 and 126 water molecules, respectively. The S1 state of a photosensitizer (Re­(bpydp)­(CO)_3_Cl, where bpydp is the 2,2′-bipyridine-4,4′-bisphosphonic acid) bound to the (101) anatase TiO_2_ surface (see Figure 4) was obtained at the computational cost comparable to the one for the DFT ground electronic state.

**Figure 4:**
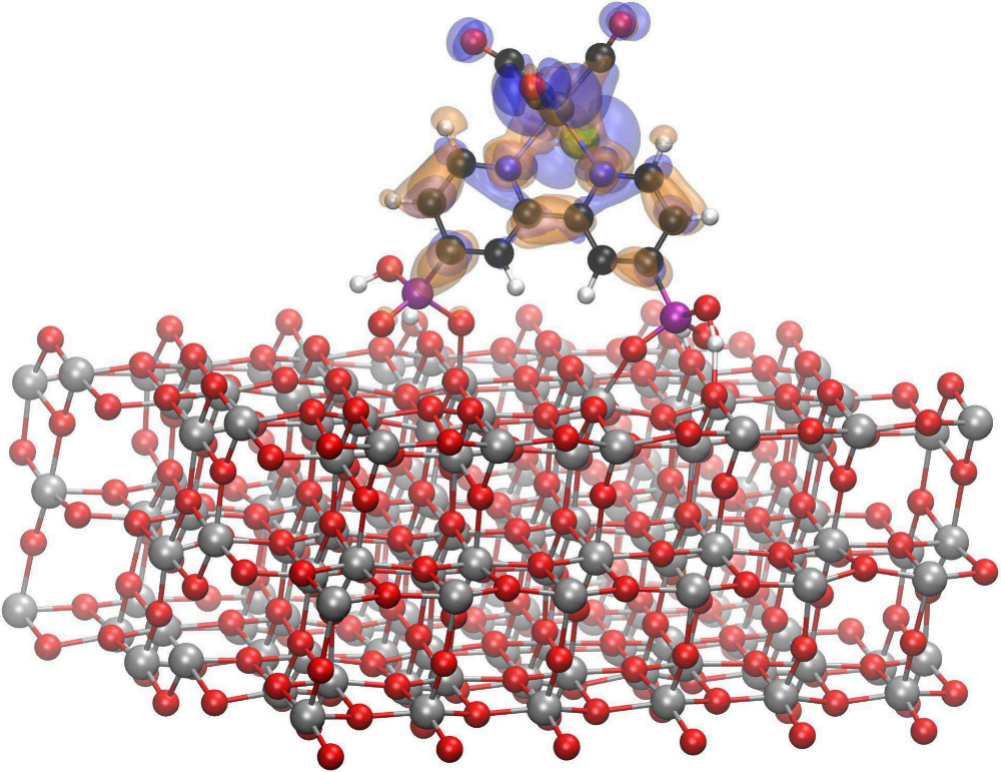
Density difference between the S1 and S0 states of the Re photosensitizer on the (101) anatase TiO_2_ surface. The blue (orange) isosurface shows the positive (negative) 0.001*a*_0_^–3^ value. Adapted from ref 61. CC BY 4.0.

##### Subsystem Density Embedding

For additional speedup of computing excited electronic states of large (periodic) systems, the Kim–Gordon (KG) subsystem density embedding (SDE) procedure was combined with the ΔSCF methodology.^115^ As with any SDE method, the total system is partitioned into smaller systems, usually not mutually bound with covalent bonds.^130–132^ For a system divided into N parts, the total electronic density, for any electronic state *i*, is also partitioned into separate subsystem electronic densities

31 $$\rho^{i}\left(r\right)=\overset{N}{\underset{s}{\sum }}\rho_{s}^{i}\left(r\right)=\overset{N}{\underset{s}{\sum }}\underset{j}{\sum }\overset{\left\{\alpha ,\beta \right\}}{\underset{\sigma }{\sum }}n_{sj\sigma }^{i}{\left|\varphi_{sj}^{i}\left(r\right)\right|}^{2}$$

where *j* is the index of KS MO. The electronic density of each subsystem is defined with corresponding occupation numbers and KS MOs. ∑_*jσ*_ *n*_*sjσ*_^*i*^ gives the total number of electrons *N*_*s*_ within a subsystem *s*, while ∑_*s*_ *N*_*s*_ = *N*_e_. Within KG SDE the total energy is defined with the Hohenberg–Kohn (HK) functional and contributions from the KS subsystems’ energies which contribute to the interaction between subsystems.^6,115^ The difference between the HK and KS energies lies in the kinetic energy term, where the former is the functional of the density, while the latter is only of MOs (see ([Disp-formula eq14])), from which an embedding potential term arises,

32 $$v_{\mathrm{emb}}\left[\rho^{i},\rho_{s}^{i}\right]\left(r\right)={\frac{\delta E_{\mathrm{kin}}^{\mathrm{HK}}\left[\rho \right]}{\delta \rho }|}_{\rho^{i}}-{\frac{\delta E_{\mathrm{kin}}^{\mathrm{HK}}\left[\rho \right]}{\delta \rho }|}_{\rho_{s}^{i}}$$

and is added to eq 22, from which the KS MOs are determined in the SCF way.^6,115^ In the system’s partitioning, the basis set is deliberately chosen to be constrained on each subsystem and the KS equation partitions into a block diagonal form, which makes the KS orbitals associated with the individual subsystem.^6,37^ This also simplifies the association of the occupation numbers since the chromophore is always a separate system from the environment.

The ΔSCF SDE implementation in CP2K was applied on the solvated diimide system.^115^ For several different partitionings of the periodic water environment (see Figure 5) extensive comparison was made with the nonembedded (NE) full system in terms of energies, their derivatives with respect to the nuclear coordinates (see Figure 6), and finally NAMD, which is further elaborated in Nonadiabatic Molecular Dynamics.

**Figure 5:**
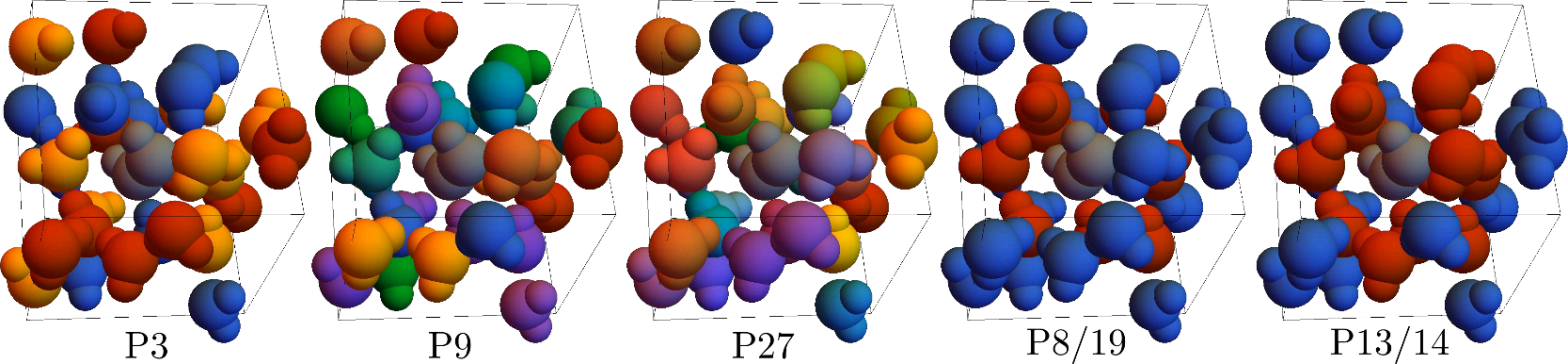
Examined partitionings of the *cis*-diimide (gray) in a periodic box with 27 water molecules. Diimide was always a separate subsystem, while the water molecules were partitioned randomly into three (P3), nine (P9), and 27 (P27) subsystems, each with an equal number of water molecules. P8/19 and P13/14 show two different partitionings of the water into inner and outer solvation shells. The inner solvation shell for the first (second) partitioning contains 8 (13) and the outer shell 19 (14) water molecules. Adapted from ref 115. Copyright 2021 American Chemical Society.

**Figure 6:**
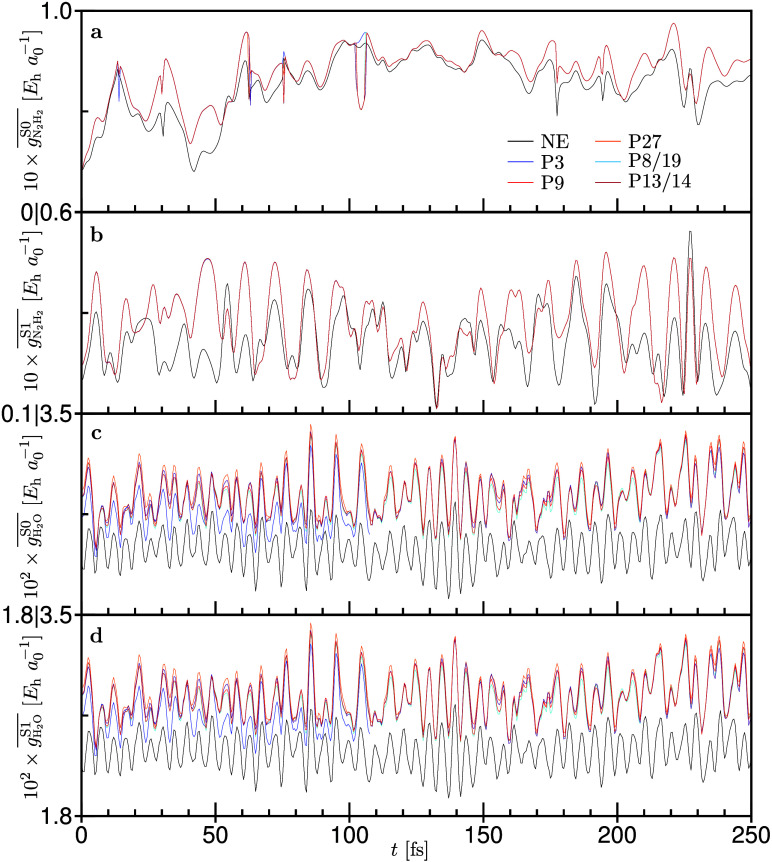
Averaged nuclear gradient norms ( $\overset{®}{g^{i}\;}=\overset{M}{\underset{j}{\sum }}\left|\nabla_{j}E^{i}\right|/M$ ) over all *M* atoms are computed for several different subsystem partitionings (P3, P9, P27, P8/19, P13/14) as shown in Figure 5. The reference values are from the nonembedded (NE) trajectory shown in black. a and b: Diimide subsystem in the ground and first singlet excited electronic states, respectively. c and d: All water molecules in the ground and first singlet excited electronic states, respectively. Adapted from ref 115. Copyright 2021 American Chemical Society.

#### 1.4 Section Summary

The ΔSCF method, as implemented in CP2K, offers a robust and versatile framework for studying excited electronic states. Multiple approaches are available, each tailored to specific needs. The original MOM enables optimization of triplet and mixed singlet–triplet states within the UKS formulation, providing an approximation for singlet excited state energies via eq 18. However, for the exact treatment of the singlet excited electronic state properties, either the ROKS SP construction or the direct construction of the singlet excited electronic state within the ROKS formulation should be applied. It should be noted that only the latter technique, i.e., ΔSCF with fractional occupation numbers, allows inclusion of multiconfiguration type excited electronic states. Two new algorithms facilitate better convergence of excited electronic states and are crucial for tracking the excited electronic state densities of multiconfigurational excited-states.^111^ Also, an OT optimization procedure for ΔSCF is available.^61^ Recently, a correction was designed that enables energy-shift free singlet excited electronic state when these states are computed with hybrid DFT functionals.^133^

These implementations have been validated against TD-DFPT and wave function-based methods. Observables such as excited electronic state energies, their nuclear gradients, TDMs, SOCs, and nonadiabatic couplings demonstrate high accuracy, making ΔSCF a reliable tool for complex systems. The methodology has been successfully applied in NAMD simulations of condensed-phase systems and shows compatibility with density embedding techniques, contributing to improved computational efficiency for large-scale calculations (see Nonadiabatic Molecular Dynamics). Altogether, the ΔSCF framework in CP2K offers a practical balance between accuracy and performance, making it a valuable asset for excited-state electronic structure simulations.

### 2 Nonadiabatic Molecular Dynamics

While a whole plethora of NAMD methods exist, the commonly used ones are based on the classical treatment of atomic nuclei and several electronic states.^134–141^ For discovering new photochemical processes, trajectory surface hopping (TSH) techniques provide direct insight into nonadiabatic (NA) processes, potentially unravelling new nonradiative deactivation (NRD) mechanisms with every trajectory. Because this methodology propagates NA trajectories independently, it fails to capture the details associated with the underlying evolution of the nuclear wave packets, so branching ratios and excited-state kinetics are not as accurate as quantum-based propagation methods, and various extensions have been proposed that mitigate certain TSH issues.^142^ Several works have reported propagating MD in excited electronic states using CP2K, such as the work by Frank and co-workers on ROKS-based spin-purified ΔSCF^22^ or RT-TDDFT Ehrenfest dynamics.^25,26^ Other studies have evolved the electronic population on excited states computed with CP2K but along predetermined nuclear trajectories.^16–18,143–147^ As no excited-state gradients were involved under the neglect of back-reaction approximation, they will not be discussed in detail in this review. For details, see references.^16–18,143–147^ To the best of our knowledge, only our previous works have performed full TSH in the condensed phase utilizing the CP2K code.^21,23,24,115^ These are, along with a brief description of the NAMD, summarized below. For a more in-depth review of ΔSCF-based NAMD, we refer the reader to reference.^88^

#### 2.1 Formalism

In TSH, a MD trajectory is propagated along one electronic state with the same state’s nuclear forces until a hop to another state occurs, and the procedure resumes in the new electronic state.^148^ Since the hop is sudden, the nuclear velocities must be rescaled to conserve the total energy, and various velocity-rescaling options have been developed.^149^ TSHs where the difference in potential energy exceeds the available kinetic energy are rejected. Simultaneously, the electronic population is propagated by evolving the time-dependent Schrödinger equation (TDSE):

33 $$i\frac{\partial C^{i}\left(t\right)}{\partial t}=E^{i}\left(R\left(t\right)\right)C^{i}\left(t\right)-i\underset{j}{\sum }D_{ij}\left(R\left(t\right)\right)C^{j}\left(t\right)$$

where the $C^{j}$ are the coefficients of the total electronic wave function $\underset{j}{\sum }C^{j}\left(t\right)\left|\Psi^{j}\left(R\left(t\right)\right)\right\rangle$ in the Born–Huang expansion.^150^ The squares of the magnitudes of the $C^{j}$ , $|C^{j}{|}^{2}$ , make up the electronic populations, which on average are consistent with the number of trajectories in the corresponding electronic state, if the decoherence is introduced in the above expression (eq 33).^142^ ***R*** (*t*) are the nuclear coordinates at time *t* along the trajectory, and the state *i* energy and the nonadiabatic coupling (NAC) term $D_{ij}\left(R\left(t\right)\right)$ are explicitly dependent on it. The latter term couples the electronic states, and can be determined with the Hammes-Schiffer and Tully’s expansion from the overlap between electronic wave functions of two consecutive points along the trajectory.^151^ Larger NACs are associated with larger probabilities of the TSH between electronic states ( $P^{i\to j}$ ), which in the case of the Tully’s fewest-switch (TFS) surface hopping algorithm^148^ is

34 $$P^{i\to j}≔\max\left(0,-\frac{C^{i*}\left(t\right)C^{j}\left(t\right)D_{ij}\left(R\left(t\right)\right)\delta t}{{\left|C^{i}\left(t\right)\right|}^{2}}\right)$$

where δ*t* is the time step used for the numerical integration of the electronic TDSE (eq 33).^152^ Another option for determining TSH probabilities is the Landau–Zener (LZ) approximation,^153–155^ which in the adiabatic representation is given by the Belyaev and Lebedev formula:^156^

35 $$P^{i\to j}=\exp\left[-\frac{\pi }{2}{\left(\frac{{\left|E^{i}-E^{j}\right|}^{3}}{\frac{\partial^{2}}{\partial t^{2}}\left|E^{i}-E^{j}\right|}\right)}^{1/2}\right]$$

To include electronic states of different multiplicities, a mixed representation is used in which the TSH probabilities between electronic states of equal multiplicities are computed via eq 34 or 35 and those between electronic states of different multiplicities by the LZ expression^157,158^

36 $$P^{Xi\to Yj}=1-\exp\left(-2\pi \frac{{\left|\left\langle \Psi^{Xi}\right|{Ĥ}_{\mathrm{SOC}}\left|\Psi^{Yj}\right\rangle \right|}^{2}}{\frac{\partial }{\partial t}\left|E^{Xi}-E^{Yj}\right|}\right)$$

where the *X* and *Y* indicate electronic state multiplicities. The one-state effective singlet–triplet SOC term ⟨Ψ^*Xi*^|*Ĥ*_SOC_|Ψ^*Yj*^⟩ is defined in eq 29.

#### 2.2 Applications

We interfaced the CP2K code with the Zagreb Surface Hopping code.^159–161^ The former conducted all the electronic structure calculations and provided the latter with the electronic energies, nuclear gradients, MOs, and the ξ terms (eq 28) for the electron–nuclear SOCs, which were, together with NACs, computed with the Zagreb Surface Hopping code. Some of the applications conducted with such CP2K-based NAMD are presented below.

The associated file-based input-output can be bypassed with a surface hopping module tightly integrated into CP2K, allowing one to write and read necessary terms directly to and from memory. This surface hopping module is currently in development and will be available in future versions of CP2K.

##### NAMD with ΔSCF

As an example, NRD mechanisms of photoexcited diimide (N_2_H_2_) in water were simulated.^23^ The S1 state was obtained with our ΔSCF method. Simulations between the initially *trans*- and *cis*-diimide forms solvated in a periodic box with 27 water molecules show different NRD kinetics. In the first 50 fs of NAMD, the *cis*-diimide form S1 state population drops below 40%, while the *trans*-diimide S1 state population is almost preserved in the same time frame (see Figure 7). Their S1 state lifetimes are identical in a vacuum. The NRD in both cases includes the torsion of the N=N bond to ∼90° where the conical intersection (CI) between S1 and S0 states is located. But because the hydrogen bonds between the N–H groups and the oxygen in water molecules are stronger and more localized in the *trans* conformation than in the *cis*, the torsion of the N=N is stiffer in the *trans* form, preventing the sudden encounter of the CI like in the *cis* conformation and therefore its faster deactivation to the ground electronic state.

**Figure 7:**
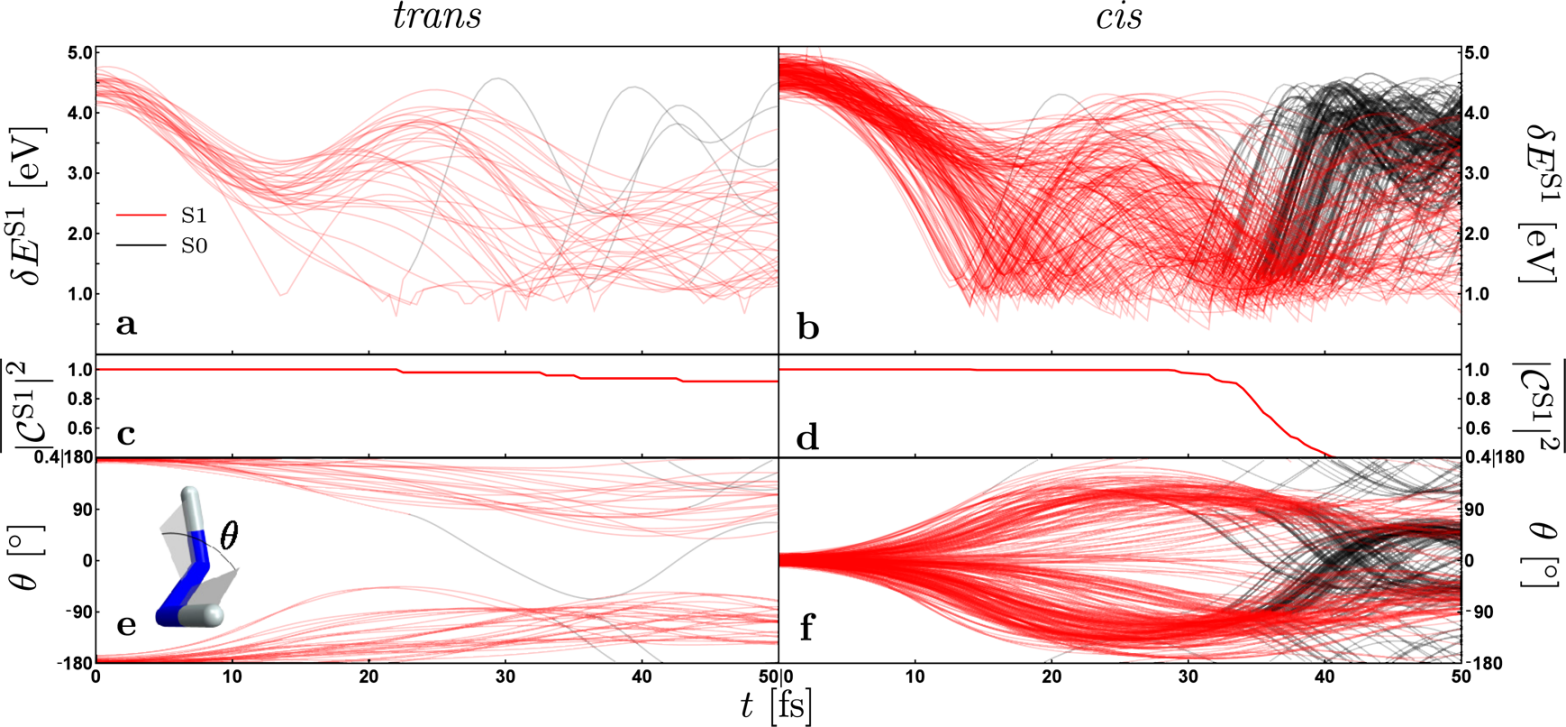
Evolution of 50 and 270 NAMD trajectories for solvated *trans*-diimide (left) and solvated *cis*-diimide (right), respectively. Only the first 50 fs of NAMD simulation are displayed. (a, b) Energy difference between the first excited and ground electronic states. (c, d) Average population of the first excited state. (e, f) Torsional angle θ between the two NH groups (see the inset of (e)). Lines in (a), (b), (e), and (f) are colored red (black) if the corresponding trajectory is in the first excited (ground) electronic state. Adapted from ref 23. Copyright 2020 American Chemical Society.

The same solvated *cis*-diimide system was systematically studied with the SDE procedure. Relevant properties for the NAMD, namely electronic state energies, nuclear gradients, and NAC terms, remained similar between different solvent partitionings (see SDE). Despite that these properties showed a more systematic deviation from the corresponding nonembedded (NE) values, the NAMD reproduced on the P9 partitioning (see Figure 5) showed almost identical NRD kinetics as the NE system. Both TFS and LZ TSH algorithms-obtained S1 state lifetimes coincided between the embedded and NE system (see Figure 8). The only significant difference between the two calculations was that the former was on average three times faster on the supercomputer we used than the latter.

**Figure 8:**
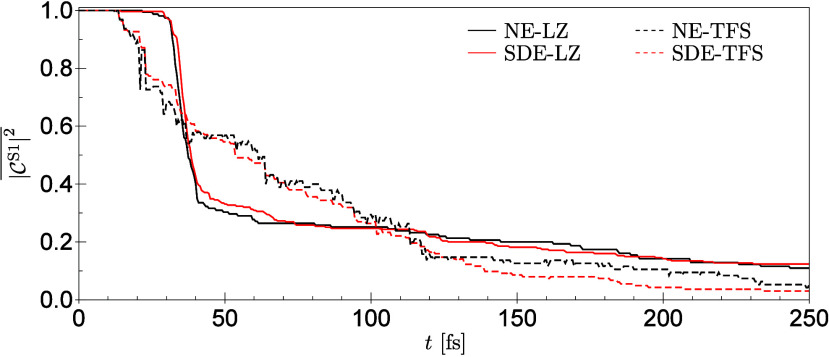
Averaged population of *cis*-diimide’s first singlet excited electronic state for the corresponding ensembles. The black solid and black dashed lines indicate the nonembedded (NE) system evolved with the Landau–Zener (LZ) surface hopping and Tully’s fewest-switching (TFS) surface hopping algorithms, respectively, while the red counterpart lines for the subsystem density embedding (SDE) with P9 partitioning of the system (see Figure 5). Adapted from ref 115. Copyright 2021 American Chemical Society.

Another example of important solute–solvent interaction during the NRD process is displayed in the cyclopropanone and its hydrate, each solvated with 25 water molecules in a periodic cubic box.^24^ Compared to simulations in a vacuum, the cyclopropanone S1 state photodissociation yield of ethylene and carbon monoxide is reduced by 18% in solution due to the solvent cage effect as the leaving CO recoils back from the first solvation shell and reforms the starting reactant. Even more interesting is the observed photochemistry of cyclopropanone hydrate where several different photoproducts are formed during its NRD (see Figure 9). The photoreaction starts with the three-membered ring opening, mostly asymmetrically, forming a carbanion in the ground electronic state after the CI that immediately reacts with the neighboring water molecule. In a few trajectories, the other C–C bond also breaks forming two or even three photoproducts. Several trajectories display a proton relocation from one site to another via a Grotthuss mechanism.

**Figure 9:**
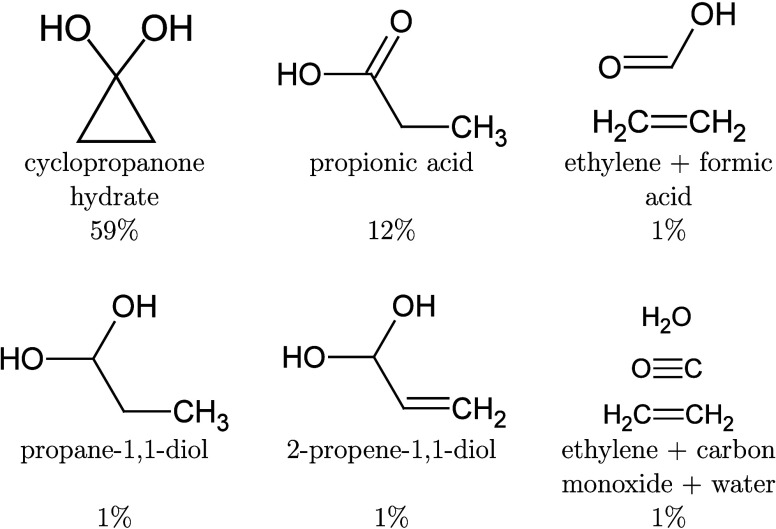
Photoproducts of the cyclopropanone hydrate NAMD calculations. The remaining trajectories decayed to the ground state, but the intermediate structures did not form a stable product by the end of the simulation time. Adapted from ref 24. CC BY-NC 3.0.

##### NAMD with TD-DFPT

An example of TD-DFPT-based NAMD with CP2K is our investigation of *o*-nitrophenol (2NP) and *p*-nitrophenol (4NP) photochemistry,^21^ where the NRD of 2NP and 4NP was modeled in the gas phase and aqueous solution, simulating atmospheric and aerosol environments. We utilized periodic TD-DFPT for both the explicitly included solvent and the solute, comparing the results with periodic QM/MM (TDDFT/MM) calculations using electrostatic embedding.^21^ All TSHs are accomplished with the LZ algorithms (eqs 35 and 36). This approach allowed for the inclusion of a large number of excited states, six for 2NP and 11 for 4NP, all of which were populated during the decay process. A significant difference was observed between the results obtained from full periodic TD-DFPT and QM/MM approaches for solvated nitrophenols, which both further differ from the simulations conducted in the gas phase. These differences are best illustrated in the state population evolutions for 4NP in Figure 10. Each trajectory was initiated from the singlet excited electronic state with the largest oscillator strength, and propagated for 250 fs. In the beginning stage of the 4NP gas phase NAMD, the population from the highly excited singlet electronic states is rapidly transferred to the lower excited electronic states in a cascade that includes intersystem crossing (ISC) to the triplet electronic states. Contrary, in the solution modeled by full TD-DFPT, the initial singlet states deactivate quickly to the S1 state from which the ISC to T1 only commences after the first 60 fs at a significantly slower rate than the internal conversion of S1 to S0 state. The solvent is the origin of this discrepancy as it hinders the intramolecular motions, reducing the size of the solute’s configuration space and therefore the available region of larger SOCs, which increase upon larger molecular deformations. In addition to the solvent-induced cage effect, the intermolecular charge transfer states also contribute to the ultrafast NRD of solvated 4NP. The results obtained with the QM/MM for 4NP are in contrast to the ones obtained with solvent at full TD-DFPT, and also to the vacuum results. While the cage effect is captured equally with the MM solvent, the polarization effect of the solvent on the excited electronic states cannot be accurately captured, as the solvent red-shifts all the excited states, reducing the gaps between all states by 0.5 eV or more. The electrostatic embedding does this up to 50% at best, due to which even the internal conversions are significantly slower. For this reason, the QM/MM population remains mostly in the S1 state at the end of the simulation. Needless to say, the intermolecular charge transfer states are also unavailable at the QM/MM level.

**Figure 10:**
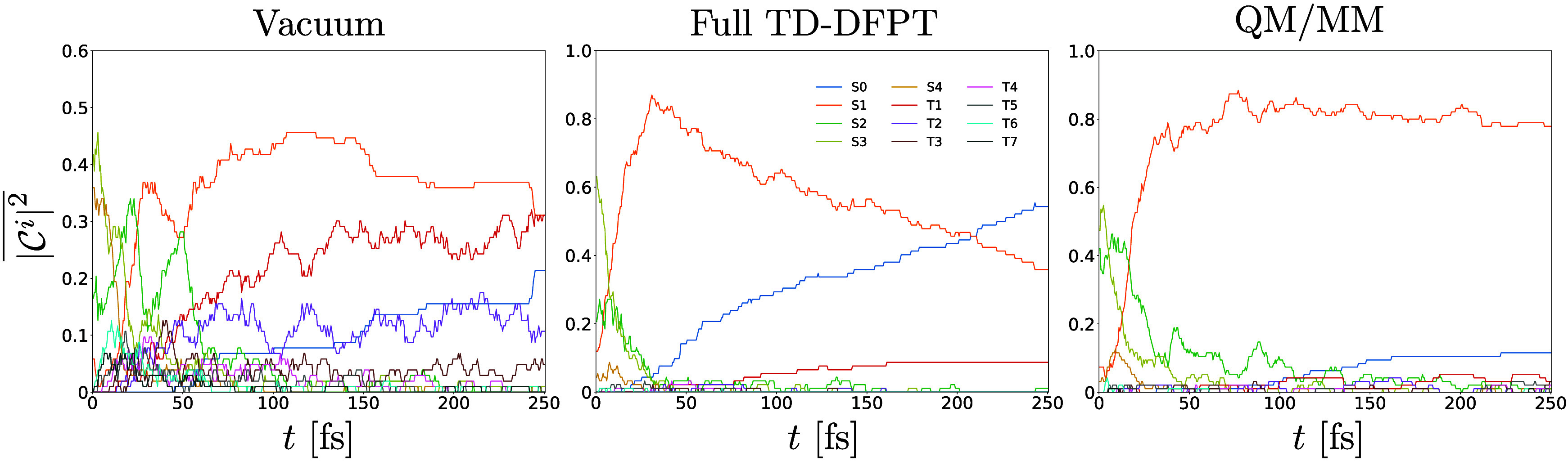
Population evolution of all electronic states in the *p*-nitrophenol (4NP) systems. Adapted from ref 21. CC BY 4.0.

Similar NRD mechanisms are displayed in the 2NP system. Given the proximity of the hydroxyl and the nitro groups in 2NP and the intramolecular charge transfer character of its S1 state, the excited-state intramolecular proton transfer (ESIPT) from the former to the latter group can generate the aci-nitro tautomer (see Figure 11). Because this tautomerisation is accompanied by a significant increase in the ground electronic state energy, ESIPT can deactivate the S1 state to the ground electronic state. ESIPT also facilitates the ISC from singlet to triplet excited electronic states. In the solvent, modeled via the full TD-DFPT, the surrounding water molecules stabilize all excited electronic states on average by 0.5 eV, increase the ESIPT barrier, and reduce the NRD. The reduced ISC in solution is in line with experimental observations.^162,163^ As the excited-state stabilization is only half at the QM/MM level compared to the full TD-DFPT, the NRD kinetics of 2NP is somewhere between the vacuum and full TD-DFPT simulations.

**Figure 11:**
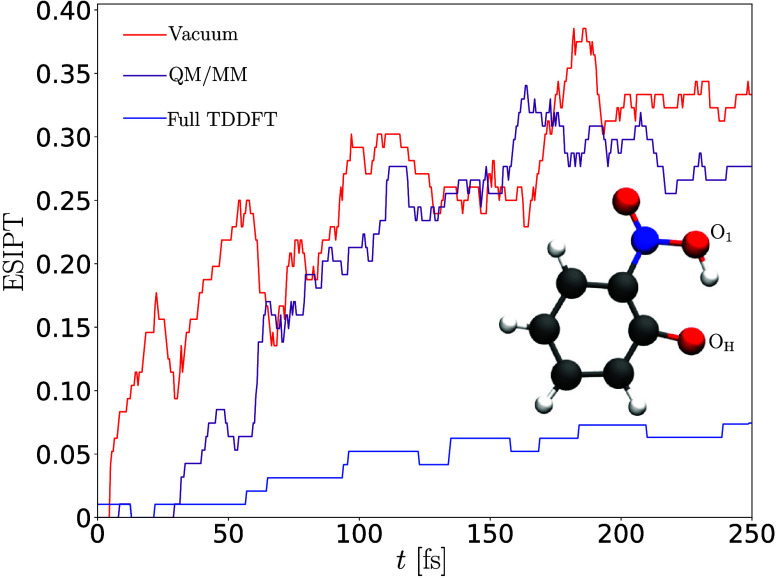
Evolution of the excited-state intramolecular proton transfer (ESIPT) in *o*-nitrophenol (2NP) trajectories, whereby ESIPT was counted if the O_1_–H bond length was greater than the O_H_–H bond length. The *y* axis indicates the occurrence of ESIPT, where 0 represents no proton transfer and 1 indicates the presence of only nitronic acid tautomers, whose example geometry is displayed in the inset. Adapted from ref 21. CC BY 4.0.

#### 2.3 Section Summary

The examples from our work demonstrate that NAMD can be performed with CP2K at the ΔSCF and TD-DFPT levels of theory. They were, to the best of our knowledge, the very first applications of these methods to NAMD in condensed-phase systems modeled by PBCs conducted with CP2K. An important observation in all results was the clear effect of solvent and the importance of modeling it at the same level of theory as the chromophore. Usually, the QM/MM method is the method of choice, due to its low computational cost, and because the most popular quantum chemistry codes rarely do excited electronic state calculations with PBCs. However, the influence this environment model exerts on the electronic states has been found inadequate, and apart from managing to capture the solvent cage effect to an adequate degree, the inability to model the direct interaction of the solute with solvent (regardless it is the polarization, hydrogen bonding, intermolecular charge transfer, or even a chemical reaction of solute with the solvent), can miss these effects and give completely different NRD mechanisms. The qualities of full NAMD simulations, where the solvent and solute are treated at equal levels, are mostly limited by the electronic levels of theory, i.e., in the case of ΔSCF and TD-DFPT. Overall, however, the ability to model such NAMD processes at the DFT level opens the possibility for modeling photochemical reactions at an all-atom level of detail not commonly available before. CP2K with its two main DFT-based electronic structure methods, ΔSCF and TD-DFPT, and particularly with its new future in situ NAMD package, will enable exactly that.

### 3 Density Functional Perturbation Theory

Density functional perturbation theory (DFPT), based on the Sternheimer equation, offers an efficient method for analytically calculating spectroscopic properties.^67,164^ In CP2K, this approach has been available for various perturbations, including electric fields, nuclear positions, nuclear velocities, and magnetic fields. These extensions enable the calculation of EPR,^39,40^ NMR,^39^ Raman spectra,^31–33,165–167^ infrared (IR) absorption spectra,^38,42^ VCD spectra^41,42^ and vibrational ROA spectra.^168,169^ Raman and ROA spectra from DFPT are limited to nonresonance spectra but (pre)­resonance effects can be included via RT-TDDFT (see ref 33) Although these methods have been implemented in MO-based response theories, their computational cost might be prohibitive for large systems. Therefore, we also explore alternative approaches such as atomic orbital (AO)-based solvers, which offer better scaling and reduced memory demands.^44,45^

#### 3.1 MO-Based Response Solver

DFPT allows to study the response of the system under study to small external perturbations. In CP2K, the implemented Sternheimer equation approach avoids direct state summation by solving the perturbed system in response to external fields. It provides an efficient way to calculate linear response properties for ground-state systems. The focus here is on the application of this formalism to time-independent calculations. The Sternheimer equation can be derived directly from the following eigenvalue equation:

37 HC=SCE

where **H**, **E**, **S**, and **C** are the KS Hamiltonian matrix, the energy eigenvalues, overlap matrix, and molecular orbital coefficients, respectively. By taking the derivative with respect to a perturbation parameter λ of an external perturbation **O**(λ), we can retrieve the Sternheimer equation in matrix form,^38,67^

38 $$S^{\left(0\right)}C^{\left(1\right)}E^{\left(0\right)}-H^{\left(0\right)}C^{\left(1\right)}=H^{\left(1\right)}C^{\left(0\right)}-S^{\left(0\right)}C^{\left(0\right)}E^{\left(1\right)}-S^{\left(1\right)}C^{\left(0\right)}E^{\left(0\right)}$$

and in the form of matrix elements,

39 $$P_{\mathrm{virt}}\left(\underset{\nu k}{\sum }\left(H_{\mu \nu }^{\left(0\right)}\delta_{kj}-S_{\mu \nu }^{\left(0\right)}E_{kj}^{\left(0\right)}\right)C_{\nu j}^{\left(1\right)}\right)=-P_{\mathrm{virt}}\underset{\nu k}{\sum }\left(H_{\mu \nu }^{\left(1\right)}\delta_{kj}-S_{\mu \nu }^{\left(1\right)}E_{kj}^{\left(0\right)}\right)C_{\nu k}^{\left(0\right)}$$

where indices *j*, *k* denote the MO coefficients and Greek symbols μ, ν represent the basis functions. The superscripts ^(0)^ and ^(1)^ denote the unperturbed and perturbed quantities, respectively. *P*_virt_ is an operator that projects onto the virtual space. Solving the Sternheimer equation calculates the contribution due to the occupied-virtual block of the MO coefficients, while the occupied-occupied contribution can be calculated separately using^170^ $C^{\left(1\right)}=-\frac{1}{2}C^{\left(0\right)}S^{\left(1\right)}$ , where **S** ^(1)^ is the overlap matrix derivative.

#### 3.2 Theory and Examples of Various Spectra

In this section, we discuss the theory of exemplary spectra calculations using DFPT.

##### Raman Spectra

Raman spectroscopy is an important technique for structural and chemical characterization. The key parameter for the Raman spectra is the calculation of the electric dipole–electric dipole polarizability tensor **α**. In the static limit, under the electric-dipole approximation, **α** describes the response of the electric dipole moment in the presence of an external electric field, and it can be written as^168^

40 $$\alpha_{\alpha \beta }=2n_{\mathrm{occ}}\underset{i}{\sum }\left\langle \varphi_{i}^{\left(1\right),\beta }\right|{\mathrm{d̂}}_{\alpha }\left|\varphi_{i}^{\left(0\right)}\right\rangle$$

where |φ^(0)^⟩ and |φ^(1)^⟩ are the unperturbed and first-order perturbed KS orbitals and *d̂*_α_ is the α component of the electric dipole moment operator. *n*_occ_ is the occupation number assuming the same numbers for all MOs. The summation over *i* runs over the number of MOs. For the nonperiodic systems, *d*_α_ = −*er*_α_, where *r*_α_ is the α component of the position operator of the electrons and *e* is the elementary charge. Polarizability calculations with RT-TDDFT are also possible; see RT-TDDFT for details.

Since this position operator is ill-defined for the periodic cases, the Berry-phase scheme^165,171^ has been employed for the calculation of polarizabilities in CP2K. Another way to calculate polarizabilities under PBC is by using the velocity form of the electric dipole moment operator, which has the form^31^

41 r^˙α=[−i∇α+i[VnlPP,r̂α]]

where ∇ is the linear momentum operator and [*V*_nl_^PP^, *r̂*_α_] is the commutator of nonlocal pseudopotentials in the KS Hamiltonian with position operator *r̂*_α_. This representation is applicable for both nonperiodic systems and periodic systems and produces consistent results with the length form of electric dipole operator and the Berry phase approach, respectively, within the complete basis set limit.^44,172,173^ Another study by Thomas et al.^174^ discusses the calculation of Raman spectra from AIMD simulations via time-correlation functions, using a finite-difference approach under an external electric field. Electric dipole polarizability calculation using maximally localized Wannier functions (MLWFs) is also compatible with PBCs and is described in ref.^169^ and decomposing the Raman intensities in terms of intra- and intermolecular contributions using an AO-based scheme is discussed in ref.^165^

In this study by Luber et al.,^165^ they also aimed at computation of Raman spectra for the liquid phase using ab initio molecular dynamics (AIMD) and comparison to static calculations of Raman spectra within the double harmonic approximation. For that, they implemented the electric dipole–electric dipole polarizability tensor using the Berry-phase scheme^175–178^ in KS-DFT. AIMD simulations under ambient conditions enable the study of system dynamics at finite temperatures, capturing effects beyond the harmonic approximation. An example of spectra calculated with AIMD and the static approach as well as an experimental spectrum are given in Figure 12.

**Figure 12:**
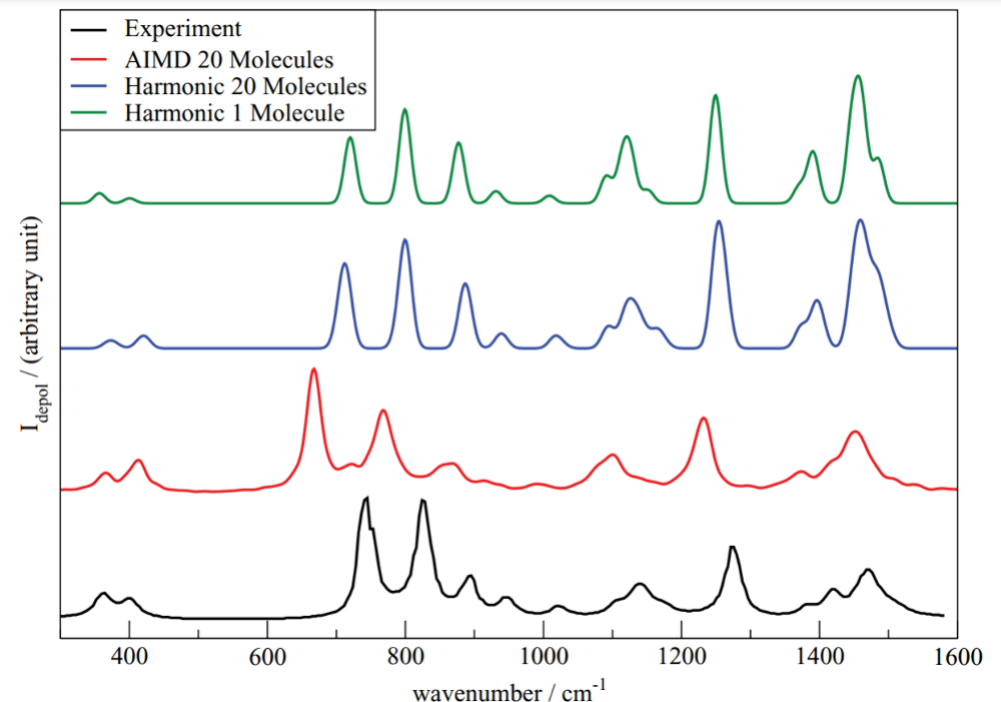
Depolarized Raman spectra of (*S*)-methyloxirane from AIMD, from static calculations in the double harmonic approximation, and comparison with experimental spectrum. The red curve represents the AIMD spectrum. All calculations used BLYP-D3/TZVP-GTH.^59,179–181^ The blue curve shows the spectrum calculated from a static calculation of a cluster of 20 (*S*)-methyloxirane molecules, obtained from a snapshot of the AIMD run. The green curve corresponds to the static spectra of a single (*S*)-methyloxirane molecule. Static Raman calculations were performed starting with the geometry optimization followed by Raman spectra calculations. The spectra are reproduced with permission from ref 165. Copyright 2014 AIP Publishing.

##### IR Absorption Spectra

IR spectroscopy is a powerful technique to provide the fingerprint of a compound, which helps in identification of molecular structure and composition by measuring their interaction with infrared radiation.^182^ The atomic polar tensors (APTs), defined as the derivative of the electric dipole moment with respect to the nuclear positions and required for static IR spectra calculations, can be calculated numerically or analytically using DFPT.^38^ For condensed systems, where the PBC are imposed on all three Cartesian directions, the Berry phase approach^165^ and the velocity form of the position operator can be used to define the electric dipole operator. MLWFs have been considered for analytical APTs as well, allowing to choose subsets of MLWFs for further analysis.^38^ Another approach has been developed based on subsystem DFT.^175^ To include finite temperature effects and simulate IR spectra under ambient conditions, AIMD simulations can also be performed in CP2K.

The recent work by Ditler et al.^38^ presents the implementation of the analytic calculation of electric dipole moment derivatives with respect to nuclear positions in CP2K, applicable to both periodic and nonperiodic systems. As an example application, two conformers of the serine-proline-alanine tripeptide were considered and the IR absorption spectra were calculated using DFPT, as shown in Figure 13. The discussion highlighted how IR spectra can distinguish between isomers based on vibrational modes and was analyzed by decomposing the system into several subsets. Additionally, they studied a periodic semiconducting system, calculating Born effective charges (BECs)^183^ and partial charges of surface atoms to understand the surface properties of anatase TiO_2_ slab and compare BECs with the reported data for bulk TiO_2_.^184,185^

**Figure 13:**
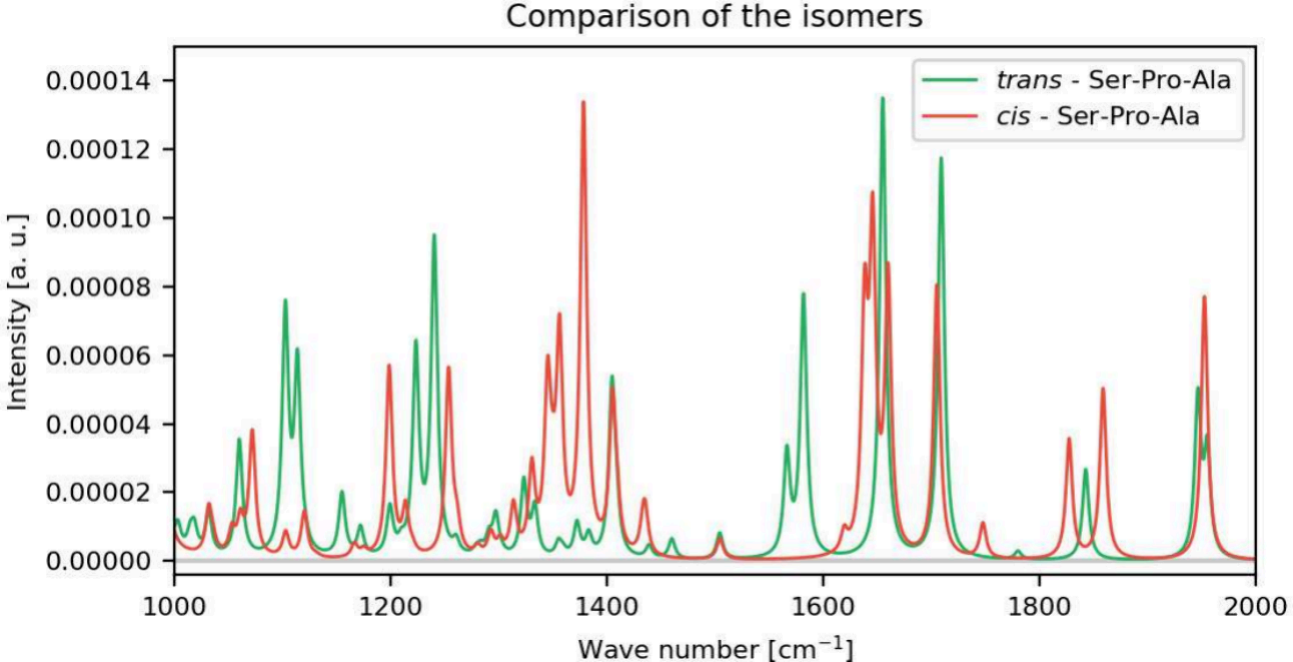
IR absorption spectra of the cis (red) and trans (green) isomers of the Ser-Pro-Ala peptide. Adapted with permission from ref 38. Copyright 2021 AIP Publishing.

##### VCD Spectra

VCD spectra are also known as IR spectra for chiral molecules,^186,187^ which are based on the interaction of circularly polarized light with chiral molecules. VCD spectroscopic technique is a measure of the differential absorption of alternating right and left circularly polarized infrared radiation. VCD spectra are theoretically characterized by the rotational strength (RS).

Theoretically, the RS for the *i*th normal mode is directly proportional to the change in electric dipole moment and magnetic dipole moment due to normal mode coordinate **Q**_*i*_:^188^

42 Ri=Im[μi·mi]

where **μ**_*i*_ = (∑_λβ_ *P*_αβ_^λ^·*S*_β,*i*_^λ^) and ***m***_*i*_ = (∑_λβ_ *M*_αβ_^*λ*^·*S*_β,*i*_^λ^) are defined by *P*_αβ_^*λ*^ and *M*_αβ_^λ^ as the APT and atomic axial tensor (AAT) elements, respectively. The Greek indices α, λ, β represent the spatial directions. *S*_β,*i*_^λ^ denotes the mass-weighted transformation matrix from Cartesian to normal mode coordinates. In the static picture, expressions of APT and AAT can be written as^41,186^

43 $$P_{\alpha \beta }^{\lambda }=\frac{\partial }{\partial {Ṙ}_{\beta }^{\lambda }}\left\langle -i\nabla_{\alpha }+i\left[V_{\mathrm{PP}}^{\mathrm{nloc}},r_{\alpha }\right]\right\rangle -\delta_{\alpha \beta }\underset{\lambda }{\sum }Z_{\lambda }$$

44 $$M_{\alpha \beta }^{\lambda ,\mathrm{vel}}=\frac{\partial }{\partial {Ṙ}_{\beta }^{\lambda }}\left\langle -\frac{r_{\alpha }\times {ṙ}_{\alpha }}{2c}\right\rangle -\underset{\lambda }{\sum }\underset{\gamma }{\sum }\varepsilon_{\alpha \gamma \beta }\frac{Z_{\lambda }}{2c}R_{\gamma }^{\lambda }$$

Here we write the electric dipole moment operator in the velocity representation, which is more convenient for periodic cases. We follow the CGS unit convention for magnetic properties. In CP2K, APTs can be calculated with the length form of the electric dipole operator using nuclear displacement perturbation theory (NDPT)^38^ as well as with the velocity form using nuclear velocity perturbation theory (NVPT)^41,42^ (for more details, see IR Absorption Spectra). In both eqs 43 and 44, the first and second terms correspond to the electronic and nuclear contributions, respectively. The derivatives are taken with respect to the nuclear velocities (*Ṙ*_β_^λ^). In NVPT, to include the nuclear-velocity-dependent gauge factor, velocity atomic orbitals are used as the basis functions.^188^ AAT calculations have been implemented using NVPT and magnetic field perturbation theory (MFPT) with gauge-including atomic orbitals (GIAOs) in the framework of DFPT.^41^ Both approaches solve response equations through the Sternheimer equation, enabling pioneering comparison of NVPT and MFPT results for VCD spectra within the same software.

The implementation was tested on a series of small molecules using LDA^46^ and BLYP^179,189^ functionals, comparing the AATs obtained from NVPT and MFPT.^41^ As can be seen in the Figure 14, all peak positions and intensities from the two theories match well for this small molecule.

**Figure 14:**
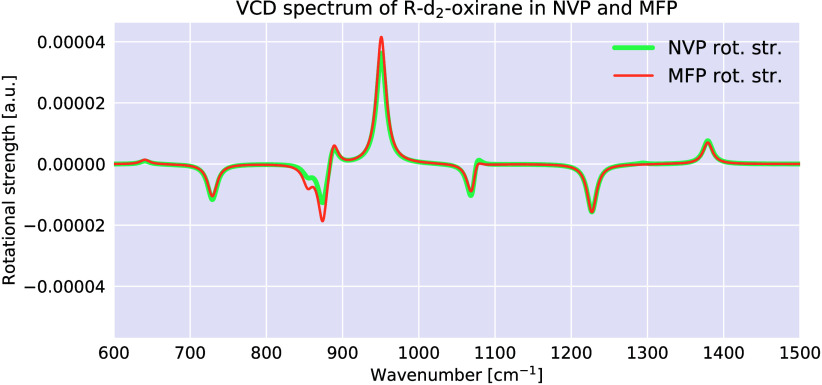
VCD spectra of *R*-*d*_2_-oxirane obtained by using the NVP and MFP atomic axial tensors. Adapted from ref 41. Copyright 2022 American Chemical Society.

Regarding the speed of RS calculations, NVPT requires 3*N* (where *N* is the number of atoms) response calculations to be performed, whereas MFPT requires only three response calculations for the three magnetic field directions. For both MFPT and NVPT AAT calculation, NDPT is required, and the coupled Sternheimer equation needs to be solved, which is the most time-consuming part. On the other hand, due to the imaginary nature of the nuclear velocities’ and the magnetic field perturbation’s Hamiltonian uncoupled equations are solved, which are less time-consuming.

Furthermore, the NVPT implementation with the GPW approach was applied to several natural products, such as limonene, carvone, pulegone, isoborneol, and camphor.^190^

##### Raman Optical Activity Spectra

ROA is a vibrational spectroscopy technique which measures the difference in Raman scattering intensity between right- and left-circularly polarized light interacting with chiral molecules. Similar to VCD spectra, standard ROA is a powerful tool for studying the structure and behavior of chiral compounds. From a theoretical perspective, ROA spectra require the calculation of the electric dipole–electric dipole polarizability tensor **α**, electric dipole–magnetic dipole polarizability tensor ***G*** ′ and the electric dipole–electric quadrupole polarizability tensor ***A***. ROA intensities are usually evaluated by taking derivatives of ROA tensors with respect to normal coordinates, in the double-harmonic approximation.^191^ Using DFT-MD simulation, the ROA intensities can be computed by the time correlation function of the ROA tensors.^168^ In CP2K, a pioneering DFPT approach has been implemented to efficiently evaluate the ROA tensors using the GPW approach. The **α** tensor calculation is discussed in Raman Spectra, while the derivation and implementation of the ***G*** ′ and ***A*** tensors in CP2K are detailed in ref.^168^

This approach was further extended to calculate ROA spectra using localized MOs, enabling the selection of appropriate subsets based on specific interests. This novel in-depth analysis allows, for instance, new insight into the complex mechanisms of how ROA intensities are generated and which part of a molecule contributes to that.^169^

ROA spectra calculation has been implemented in CP2K for both static and DFT-MD approaches. The first ROA spectra from DFT-MD (gas-phase molecule (*S*)-methyloxirane^168^) are shown in Figure 15. Additionally, the first simulation of ROA spectra for liquids including non-, pre-, and on-resonance effects using PBCs and RT-TDDFT is discussed in ref 36.

**Figure 15:**
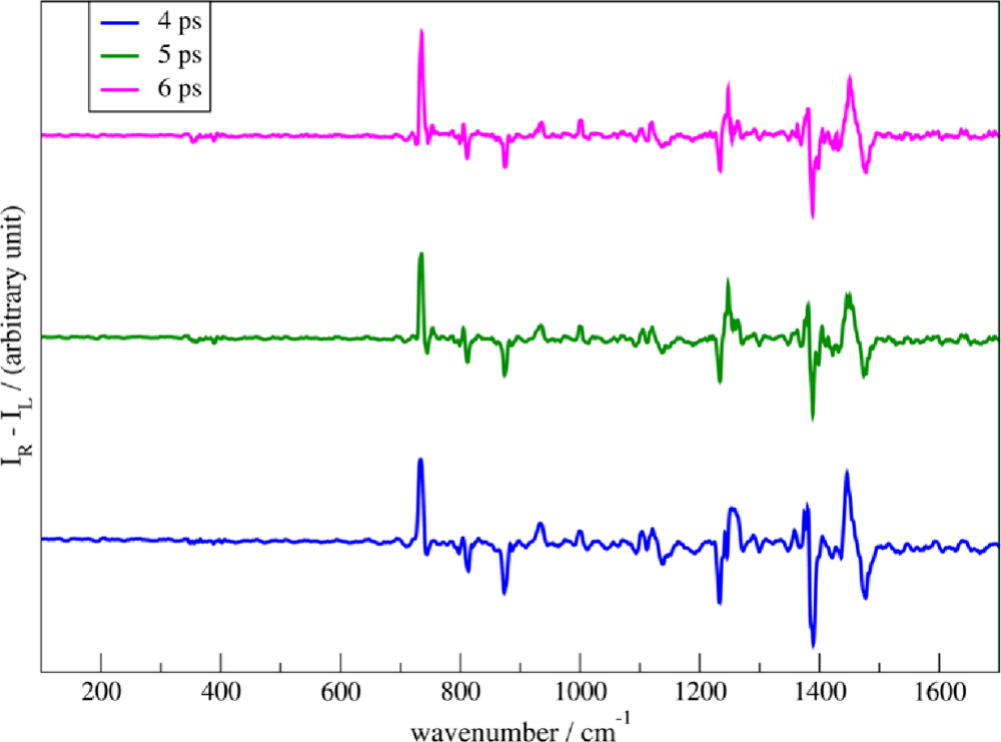
Backscattering ROA spectra of (*S*)-methyloxirane from DFT-MD simulations. The spectra were calculated with simulation lengths of 4, 5, and 6 ps. Reproduced from ref 168. Copyright 2017 American Chemical Society.

##### Sum Frequency Generation

Vibrational sum frequency generation (SFG) is a second-order nonlinear technique which enables us to investigate surface-specific signals for non centrosymmetric crystals and interfaces, thus allowing to study binding motifs of molecules on (crystal) surfaces. Within the electric dipole approximation the intensity of the SFG signal is given by^192^

45 $$I^{\mathrm{SFG}}\propto {\left|\chi_{\mathrm{eff}}^{\left(2\right)}\right|}^{2}I^{\mathrm{vis}}I^{\mathrm{IR}}$$

where *I* ^vis^ and *I* ^IR^ are the intensities of the laser pulse in the visible and IR range, respectively, which overlap at the surface. χ_eff_^(2)^ is the effective second-order nonlinear susceptibility describing the SFG process and can be approximated using DFPT. Neglecting the frequency dependence of the Fresnel coefficients, for a certain experimental setup described in ref 192 and using a (110) rutile surface (*C*_2*v*_ symmetry), the effective second-order nonlinear susceptibility can be expressed as the Fourier transform of the autocorrelation of the electric dipole–electric dipole polarizability **α** and the total electic dipole moment ***d***:

46 $$\chi_{\mathrm{eff},\mathrm{ssp}}\propto i\int_{0}^{\infty }dt\exp\left\{i\omega t\right\}\left\langle \alpha_{yy}\left(t\right)d_{z}\left(t\right)\right\rangle$$

The expectation values of α_*yy*_(*t*) and *d*_*z*_(*t*) are collected along a DFT-MD trajectory.

Using CP2K and efficient DFPT for the calculation of **α**, this method was applied to compute the first SFG spectrum for a gas–solid interface from DFT-MD, namely, the SFG response of acetonitrile molecules on a rutile (110) surface (Figure 16a). The corresponding SFG spectrum is shown in Figure 16b.

**Figure 16:**
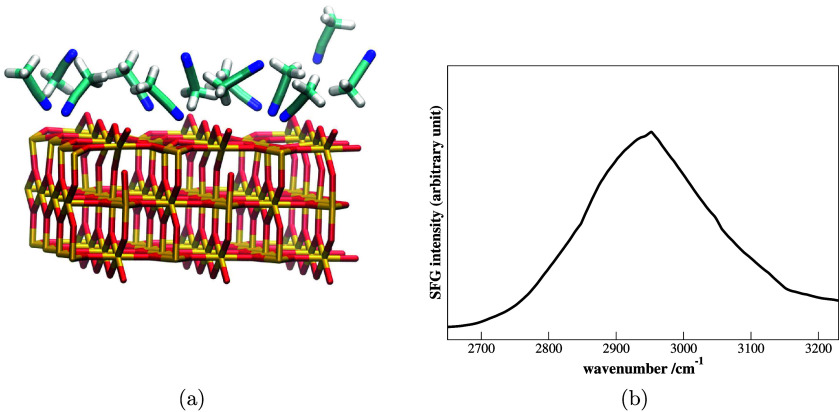
(a) Rutile (110) surface with adsorbed acetonitrile molecules. (b) Corresponding SFG spectrum calculated with DFT-MD. Reproduced from ref 192. Copyright 2016 American Chemical Society.

#### 3.3 Using the AO-Based Response Solver

The AO-based response solver serves as an alternative to the commonly used MO-based solver for studying the response of electronic systems to external perturbations. CP2K has recently incorporated the AO-based linear response solver to compute the perturbed density matrix.^43^ This solver utilizes an exponential parametrization scheme introduced by Helgaker et al.^193^ to optimize the single-electron density matrix. The response equations in AO-based theories require the KS Hamiltonian, the AO density matrix, and the overlap matrices, all of which are highly sparse. This sparsity makes the AO-based approach well-suited for large-scale simulations, where the computational cost and memory demands are critical considerations. Moreover, the AO-based formalism avoids the explicit construction of MO coefficient matrices, enabling efficient parallelization with linear-scaling techniques. As a result, this method provides a scalable and robust framework for computing response properties in both nonperiodic and periodic systems. Advantages of employing the AO-based solver thus include the following:

- *Efficiency for large systems:* Due to the highly sparse nature of the matrices in AO-based theory, these operations can be executed efficiently using the DBCSR matrix library in CP2K.^194^ That leads to improved computational efficiency for large systems over MO-based theory.^43^
- *Reduced memory requirements:* It has been observed^44^ that the sparse matrix operations in AO-based theory significantly reduce memory requirements for response calculations. In contrast, MO-based linear response methods involve MO matrices and can lead to higher memory demands and slower computations, especially for larger systems.

#### 3.4 Spectroscopic Calculations Using the AO-Based Solver

Details of the AO-based response equations for calculating the response density matrix are provided in refs 43, 44, and 195. We conducted a comparative analysis between the AO- and MO-based solvers in ref 44, which has been extended to obtain response density matrices for perturbations such as electric field, nuclear displacement, nuclear velocity, and magnetic fields,^45^ enabling the calculation of electric dipole–electric dipole polarizability tensors, APTs using NDPT, APTs and AATs from NVPT, and AATs from MFPT. As anticipated, the properties computed using the AO-based method are in line with those obtained from the previously implemented MO-based calculations.^44^

Electric dipole–electric dipole polarizability tensor calculations were implemented using AO solver for nonperiodic and periodic (only at the Γ point) systems. Parallelization significantly accelerated the AO-based solver for a test system of 128 water molecules on the specific supercomputer used, though the MO-based solver remained faster for this system size. For larger systems (up to 4096 molecules), the AO-based solver calculations were advantageous, demonstrating better scaling and potentially reduced memory demands as system size increased.^44^

The calculated VCD spectra using both AO- and MO-based solvers yield consistent results, as shown in Figure 17.^45^

**Figure 17:**
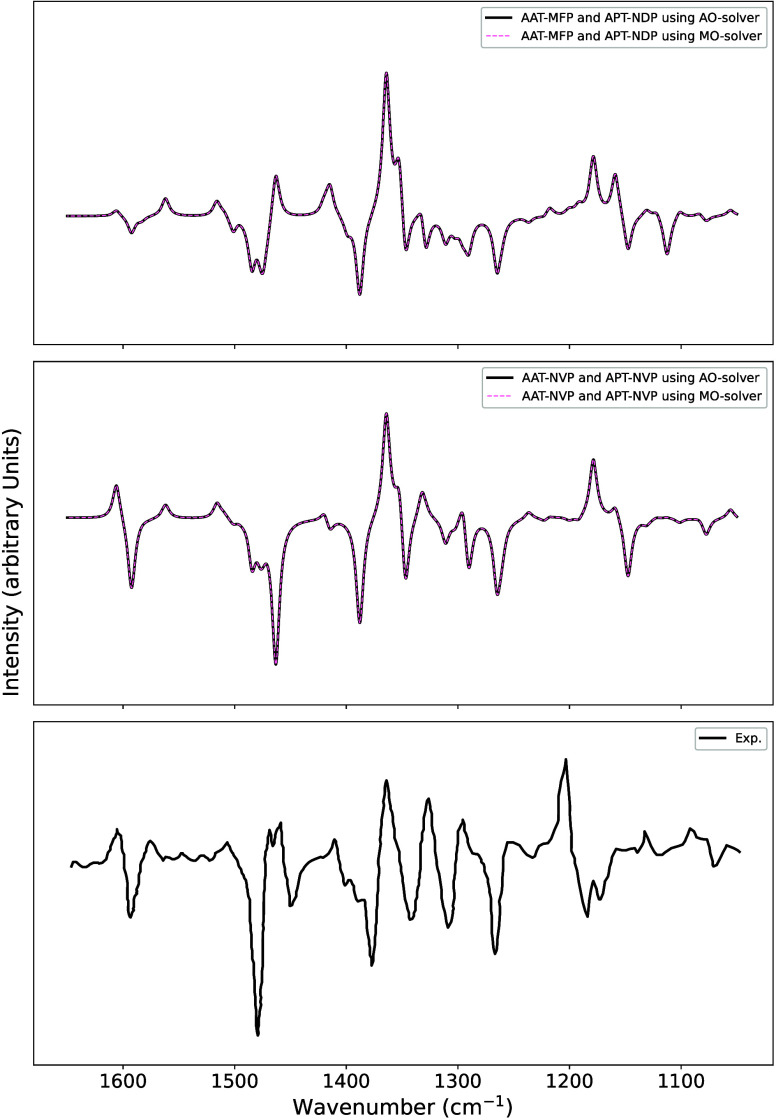
Simulated VCD spectra of R-enantiomer of mirtazapine (C_17_H_19_N_3_) based on NVPT and MFPT approaches using MO- or AO-based response solver. Top panel: AAT-MFP from MFPT and APT from NDPT; middle panel: AAT-NVP and APT-NVP both from NVPT. Adapted from ref 45. CC BY 4.0.

#### 3.5 Section Summary

DFPT in CP2K provides a reliable and efficient framework for computing a broad range of spectroscopic properties with high accuracy. Based on the Sternheimer equation, it supports the analytical evaluation of various spectroscopic responses, including NMR, EPR, Raman, IR, VCD, ROA, and SFG spectra, in periodic (at the Γ point) and/or nonperiodic systems. VCD is available in a unique fashion from both NVPT and MFPT. The implementation of MO-based and AO-based solvers offers flexibility for systems of different sizes. In particular, the AO-based solver offers better scaling for large systems compared to the MO-based solver, with reduced memory requirements and improved parallel performance. These developments expand the scope of DFPT to study complex systems under realistic conditions, including those requiring PBC and AIMD, providing outstanding opportunities for a realistic modeling of spectra.

### 4 Real-Time Time-Dependent Density Functional Theory

The Runge–Gross theorem shows that RT-TDDFT works on nonperturbative dynamics,^196–200^ but in this review we focus on spectrum calculations in external fields of perturbative strength. Spectroscopic applications of RT-TDDFT were pioneered by Yabana and Bertsch.^201^ They established a method to calculate optical spectra in a RT formulation. This technique has been extended to include solids,^202^ core-level X-ray absorption spectra^203^ electronic circular dichroism (ECD),^31,204–207^ and resonance Raman spectroscopy,^33^ among others.

RT-TDDFT spectra have been reported to be in good agreement with experimental results.^201,202^ In general, however, the accuracy is dependent on the choice of the xc functional and there is no systematic improvement, which is in contrast to multiconfiguration wave function approaches and Green’s function approaches with many-body effects included in the form of self-energy.^208^ In this review, we work on semilocal xc functionals, but hybrid functionals^128,189,209–211^ and long-range-corrected functionals^212^ with exact-exchange interactions have also been used^213^ in the literature. RT-TDDFT is commonly used in the adiabatic approximation, neglecting the dependence of the time-dependent exchange–correlation functional on the history and the starting point by using the instantaneous density in functionals designed for (stationary) ground states. This approximation can lead to spurious effects which become more pronounced if strong fields are used in RT-TDDFT.^214^

In general, RT-TDDFT is more suited for large systems with a high density of states and/or calculation of a wide energy range^215,216^ because of its favorable scaling to the system size. RT-TDDFT calculates properties of a superposition of excited states in the time-dependent wavepacket formulation, hence it is more suited for calculations of optical spectra that include excitation energies of multiple states. The RT formulation also realizes inclusion of (pre)­resonance effects for vibrational spectra.

#### 4.1 Formalism

In this section, we discuss the RT-TDDFT approach in the Kohn–Sham^47^ formulation. It was shown^217,218^ that under certain conditions (see ref 218 for details), the dynamics of many-electron systems are mapped onto those of effective noninteracting systems,

47 $$i\frac{\partial }{\partial t}\varphi_{i}\left(r,t\right)=\left(\mathrm{T̂}+v_{\mathrm{ext}}\left(r,t\right)+v_{H}\left(r,t\right)+v_{\mathrm{xc}}\left(r,t\right)\right)\varphi_{i}\left(r,t\right)$$

where *T̂* is the kinetic energy operator and *v*_ext_(**r**, *t*), *v*_H_(**r**, *t*), and *v*_xc_(**r**, *t*) are the external, Hartree, and exchange–correlation (xc) potentials, respectively. The exact time-dependent xc potential is not known, and hence, the calculation results are of limited accuracy dependent on the choice of the xc functional. We here assume the validity of adiabatic approximation and use known xc functionals for the ground state (LDA,^219^ GGA,^127,220^ etc.) evaluated at the time-dependent density ρ­(**r**, *t*). Additionally, for calculations of periodic systems in this review, we only consider semilocal xc functionals.

In a spectroscopic response calculation with RT-TDDFT, the electromagnetic fields are included explicitly in the simulation run during the time propagation, often only at the beginning, i.e., in general the response of operator *B̂* with respect to a perturbation according to *Âf*(*t*) is constructed during the propagation run. In the time domain, the linear response is given by

48 $$\left\langle \mathrm{B̂}\left(t\right)\right\rangle -{\left\langle \mathrm{B̂}\right\rangle }_{0}=\int_{-\infty }^{t}dt^{\prime }\left\langle \left\langle \mathrm{B̂};Â\right\rangle \right\rangle \left(t-t^{\prime }\right)f\left(t^{\prime }\right)$$

where ⟨⟨*B̂*; *Â*⟩⟩ denotes the linear response function of the response of *B̂* with respect to *Â*, *f*(*t*) denotes the functional form of the electric field or the vector potential, and it is assumed that the perturbation is switched on at *t* = 0. The linear response function in the energy domain ⟨⟨*B̂*; *Â*⟩⟩_ω_, with angular frequency ω, is then given as

49 $$\left\langle \mathrm{B̂}\left(\omega \right)\right\rangle =\langle \left\langle \mathrm{B̂};Â\right\rangle \rangle_{\omega }f\left(\omega \right)$$

where

50 $$\left\langle \mathrm{B̂}\left(\omega \right)\right\rangle =\underset{\varepsilon \to 0^{+}}{\lim}\int_{-\infty }^{\infty }dt\left(\left\langle \mathrm{B̂}\left(t\right)\right\rangle -\langle \mathrm{B̂}\rangle_{0}\right)e^{i\omega t}e^{-\varepsilon t}$$

where ε is an infinitesimal positive number to be taken to the limit ε →0^+^ and can thus be constructed from the Fourier transform of the response during the RT-TDDFT run because the form of the applied field is known. In practice often a δ pulse is used for the electric field, which excites the full spectrum of the molecule. As such, the perturbing field can be applied explicitly or using DFPT in the occupied–virtual space of the optimized ground state.^32^

#### 4.2 Implementation Details in CP2K

The implementation in CP2K simply proceeds by integrating the time-dependent Kohn–Sham equations (eq 47) for small time steps, usually on the order of attoseconds (∼1 au). Due to the Nyquist theorem,^221^ the time step determines the highest resonance that can be sampled during an RT-TDDFT simulation. Thus, apart from the numerical stability of the integrator, the largest excitation energy of the system sets a limit on the maximum size of the time step.

The approximations of the integrator are twofold: the finite difference of the integrator itself and an approximation of the matrix exponential involved. In CP2K, the following integrators are implemented:^25^

1. Enforced time reversible symmetry (ETRS);
2. Crank–Nicholson;^222^
3. Exponential midpoint rule.

The exponential in the propagator may be calculated by the following methods:

1. Taylor or Pade expansion;
2. Arnoldi subspace algorithm;^223^
3. Baker–Campbell–Hausdorff expansion.^224–226^

It is possible to propagate either the MO coefficients or the density matrix directly.^25^ The propagator may be calculated self-consistently up to a specified precision in order to achieve more stable propagation runs.

The RT-TDDFT implementation is easily extended to EMD by allowing the nuclei to move during the simulation.^25^

In this section, Greek indices (α, β, ...) represent the spatial directions (*x*, *y*, *z*).

##### Nonperiodic Systems

Recently the RT-TDDFT implementation was extended to simulate several kinds of spectrocopies in the linear response regime.^31–34,166^ Specifically, the inclusion of calculations in the velocity gauge (VG) as well as magnetic response properties are included. For the GPW method, including pseudopotentials, special care has to be taken to use the correct definition of the velocity operator because the nonlocal part of the pseudopotentials does not commute with the Hamiltonian. Thus, the velocity operator in eq 41 ^32,166,167^ is used in the following.

In order to extend the functionality of the RT-TDDFT implementation with respect to spectroscopic simulations, the following operators are implemented:

- Electric dipole moment: length (−**r̂**) and velocity representation (−**r̂̇**).
- Magnetic dipole moment: l̂=−12r̂×r^˙ .
- Electric quadrupole: length (*q̂*_αβ_ = – *r̂*_α_ *r̂*_β_) and velocity representation (*q̂*_αβ_^vel^ = – (*r̂*_α_ *r̂̇*_β_ – *r̂̇*_α_ *r̂*_β_).

For spectroscopic simulations with RT-TDDFT in CP2K, the Coulomb gauge is chosen, and in the dipole approximation its special cases length gauge (LG) (where the vector potential is chosen to be zero) and VG (where the scalar potential is chosen to be zero) are used. In the case of a δ pulse, VG yields a constant vector potential,^227^ which requires an explicit gauge transformation of the nonlocal pseudopotential part if the vector potential is taken into account nonperturbatively.^32^

The extension of the RT-TDDFT implementation in CP2K is summed up in Table 1, indicating which operators are available as perturbation operators and which as response operators. The nonlocal commutator for the definition of the velocity operator is used wherever applicable. With this set of operators available, it can be used as a toolbox to calculate different kinds of response functions (see eq 49) required for the simulation of specific spectroscopic experiments.

**Table 1:** Operators Available for Perturbation and Response in an RT-TDDFT Calculation with CP2K

	electric dipole	magnetic dipole	electric quadrupole
perturbation	**r̂**, **r̂̇**	**l̂**	
response	**r̂**, **r̂̇**	**l̂**	**q̂**, **q̂**^vel^

##### Periodic Systems without **k**-Point Sampling (Γ-Point Calculations)

In periodic systems, the external field term in the LG formulation **r̂**·**E**, where **E** is the electric field strength, is not applicable since it breaks the lattice periodicity. In the case of spatially uniform external fields, the gauge transformation to the VG formulation, $\varphi_{ki}^{\left(\mathrm{LG}\right)}\left(r\right)=e^{iA\cdot r/c}\varphi_{ki}^{\left(\mathrm{VG}\right)}\left(r\right)$ with the vector potential **A** = – *c*∫^*t*^ d*t*′ **E**(*t*′), converts the KS Hamiltonian to a lattice-periodic form:^202,228^

51 $$H^{\mathrm{KS}}\left(r,r^{\prime }\right)=\frac{1}{2}{\left(\frac{\nabla }{i}+\frac{1}{c}A\right)}^{2}\delta^{3}\left(r-r^{\prime }\right)+e^{-iA\cdot r/c}V\left(r,r^{\prime }\right)e^{iA\cdot r\prime /c}$$

Another problem arising from PBCs relevant for spectroscopic applications is that the observables containing the position operator **r̂** become ill-defined in periodic systems, as we discussed earlier in the DFPT section. For electronic dipoles, one can transform them into well-defined operators (eq 41), which is also valid in periodic systems. Alternatively, one can apply the modern theory of polarization^176,229^ to get well-defined electric polarization in periodic systems. These position operators for spectroscopic applications in periodic boundary conditions are discussed in detail by Ditler et al.^42^

##### Localized Orbitals in RT-TDDFT

In the Bloch picture, an MO is a Fourier series of real-space plane waves.^230^ There exists a unitary transformation ***U*** which leaves the observables of the MO unchanged but localizes the MO into a single cell of the periodic lattice:^231,232^ The resulting transformed set of MOs are MLWFs or, more generally, the set of localized orbitals (LOs).

The transformation matrix ***U*** ^ml^ can be found by numerical optimization with any simultaneous diagonalization algorithm, most commonly the Jacobi rotation algorithm.^233^ Since they are usually limited to real-valued matrices and are thus not applicable to time-dependent MOs (which are complex-valued), we have implemented the Jacobi rotation algorithm with Cardoso–Souloumiac rotation angles.^35,234^

CP2K offers the Foster–Boys criterion,^235^ which uses the second moment of the MOs, as well as the spread functional of Berghold et al.^236^ which is applicable to PBCs. Since these spatially based spread functionals do not take symmetry into account, they are prone to σ/π symmetry mixing, resulting in so-called “banana bonds” (see Figure 18b). This can be circumvented by the use of symmetry-preserving spread functionals, such as the partial-charge-based Pipek–Mezey criterion (see Figure 18c).^237,238^ The propagated localization method in CP2K implements this functional as well.^239^

**Figure 18:**
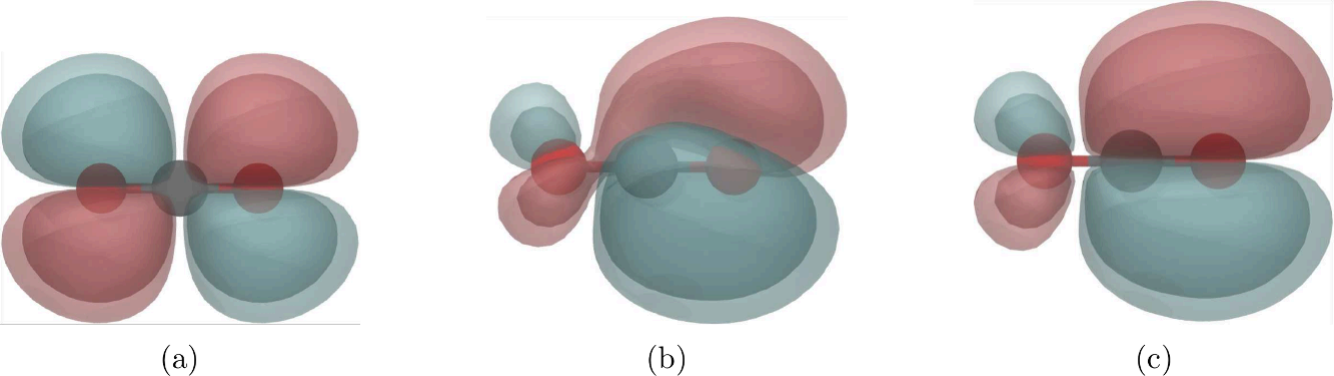
Examples of localization: (a) canonical three-center HOMO, (b) the corresponding Foster–Boys Wannier function, showing a typical “banana bond”, and (c) the Pipek–Mezey Wannier function, showing symmetry-preserving LO. Reproduced from ref 239. CC BY 4.0.

With the help of LOs, it is possible to separate periodic systems into localized subsystems, and treat their LOs with distributed gauge origins. This procedure was previously demonstrated with Kim–Gordon subsystem DFT as a means of subsystem separation.^175^ It can also be applied to operators which are typically ill-defined in PBCs, most importantly the magnetic dipole and electric quadrupole moment operators in VG.^36^

Using MLWFs, the *local circulation* ^240^ part of the magnetic moment per unit cell is calculated as^240^

52 $$\left\langle {\mathrm{m̂}}_{\alpha }\right\rangle =-\frac{1}{2c}\underset{i}{\sum }\left\langle w_{i}\right|\underset{\beta \gamma }{\sum }\varepsilon_{\alpha \beta \gamma }{\mathrm{r̂}}^{\beta }{\mathrm{p̂}}^{\gamma }\left|w_{i}\right\rangle$$

where *w*_*i*_ represent the MLWFs in the unit cell at the origin. The remaining part, the *itinerant circulation* ^240^ part, was regarded as irrelevant in calculations in reference,^36^ where we assumed a large unit cell and vanishing surface current. For the Γ-point limit, the expectation value of the electric dipole moment is usually calculated via the Berry phase approach as^178^

53 ⟨d̂α⟩=noccLα2π⁡Im⁡ln⁡det⁡Sα

where *L*_α_ is the side length of the simulation cell in the αth direction (assuming a rectangular cell), *n*_occ_ are the occupation numbers, which we assume to be the same for all MOs, and the elements of the matrix *S*_α_ are given in terms of the KS orbitals φ_*i*_ (assuming real KS orbitals) as

54 $$S_{\alpha ,ij}=\left\langle \varphi_{i}|\exp\left\{-\frac{2\pi i}{L_{\alpha }}{\mathrm{r̂}}_{\alpha }\right\}|\varphi_{j}\right\rangle$$

Luber^37^ and Schreder and Luber^35^ showed that the eq 53 can be approximated to first order in the MLWF basis as

55 ⟨d̂α⟩≈−noccLα2π⁡Im⁡ln⁡exp(i2πLα∑i⟨wi|r̂α|wi⟩)

The electric quadrupole moment is calculated in an analogous manner as^36^

56 ⟨q̂αβ⟩≈−noccLαLβ2π⁡Im⁡ln⁡exp(i2πLαLβ∑i⟨wi|r̂αr̂β|wi⟩)

These techniques were applied for spectroscopic calculations of solvated molecules with PBCs.^35,36^ In these calculations, which were carried out in large unit cells, the Γ-point limit of the modern theory of polarization was used to obtain the expectation value of the electric dipole moment.^178^

##### Periodic Systems with **k**-Point Sampling

RT-TDDFT with **k**-point sampling has recently been developed,^241^ featuring an efficient **k**-point parallelization scheme. It also includes an extension to DFT+U,^242–244^ using a robust LO projection scheme developed by O’Regan et al.^245^ and Chai et al.^246^ DFT+U is a cost-efficient method that allows applications to strongly correlated systems, in which semilocal xc functionals such as LDA or GGA lead to qualitatively wrong predictions of properties due to the self-interaction error.

In lattice periodic systems where the KS Hamiltonian satisfies *H* ^KS^(**r** + **T**_*n*_) = *H* ^KS^(**r**) with a lattice translation vector **T**_*n*_, the KS orbitals are Bloch functions characterized by Bloch vectors **k** and band indices *i* and satisfy φ_**k** *i*_(**r** + **T**_*n*_) = e^i**k**·**T**_*n*_^φ_**k** *i*_(**r**). The time-dependent KS eq 47 is integrated for each **k** vector in an analogous manner to nonperiodic systems. Details of the formulation are given in ref 241.

#### 4.3 Applications

##### Nonperiodic Systems

The extension of the RT-TDDFT implementation described in Nonperiodic Systems allows a variety of linear response tensors which are relevant for spectroscopic experiments to be obtained. In particular, the following response tensors are available for different representations (here given in LG):

57 $$\alpha_{\alpha \beta }\left(\omega \right)=-\langle \left\langle {\mathrm{d̂}}_{\alpha };{\mathrm{d̂}}_{\beta }\right\rangle \rangle_{\omega }$$

58 $$G_{\alpha \beta }\left(\omega \right)=-\langle \left\langle {\mathrm{m̂}}_{\alpha };{\mathrm{d̂}}_{\beta }\right\rangle \rangle_{\omega }$$

59 $$G_{\alpha \beta }\left(\omega \right)=-\langle \left\langle {\mathrm{d̂}}_{\alpha };{\mathrm{m̂}}_{\beta }\right\rangle \rangle_{\omega }$$

60 $$A_{\gamma ,\alpha \beta }\left(\omega \right)=-\langle \left\langle {\mathrm{q̂}}_{\alpha \beta };{\mathrm{d̂}}_{\gamma }\right\rangle \rangle_{\omega }$$

With these tensors, the spectroscopic invariants for UV/vis absorption,^33^ electronic circular dichroism,^31,32^ Raman,^33,166^ and Raman optical activity (ROA)^34^ can be calculated for different choices of gauge. The vibrational degrees of freedom for Raman and Raman optical activity can be treated either in the harmonic approximation where the response tensors are expanded in terms of normal modes, or in a time-dependent picture using time autocorrelation functions of the tensors.^166^ The main advantage of using RT-TDDFT to simulate these spectroscopy lies in the treatment of non-, pre-, and resonance cases on equal footing, employing the short time approximation in the resonance case. The resulting excitation profiles are illustrated for the Raman profile (Figure 19a) and the ROA profile (Figure 19b) of (*R*)-methyloxirane.

**Figure 19:**
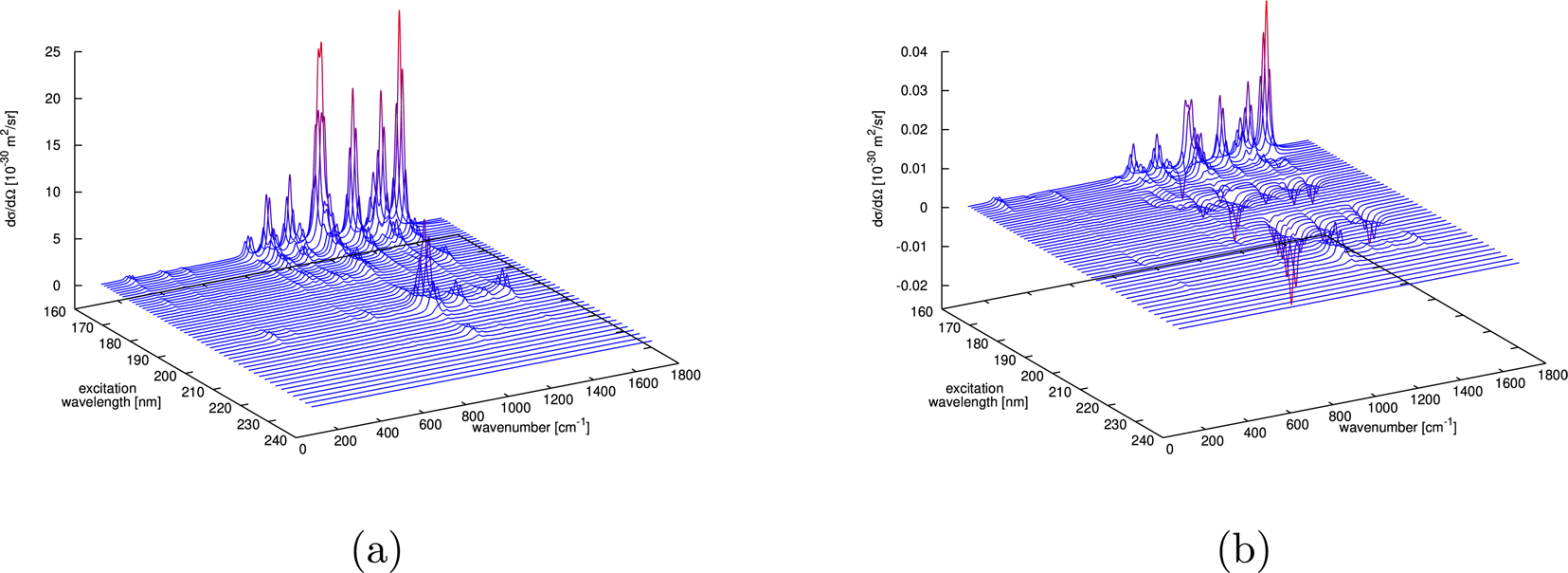
Raman (a) and ROA (b) excitation profiles of (*R*)-methyloxirane. Adapted with permission from ref 34. Copyright 2019 AIP Publishing.

RT-TDDFT is a valuable tool to investigate the gauge origin dependence of TDDFT calculations. In the linear response regime it gives the same results as a TD-DFPT calculation.^247^ However, in contrast to TD-DFPT, RT-TDDFT gives a more intuitive understanding of the choice of gauge as the electric field and vector potentials are applied explicitly, forcing a certain choice of gauge to be explicit in the calculation. In the cases where magnetic response properties become important, such as ECD or ROA, only certain combinations of perturbation and response operators give gauge independent results.^248^

##### Periodic Systems without **k**-Point Sampling

RT-TDDFT obeying PBCs without (multiple) **k**-point sampling, typically carried out at the Γ point, has been applied to study solvent effects in spectra^35,36,249^ in order to fully reproduce solute–solvent interactions by including excitations of solvent as well as solute molecules. Schreder and Luber^35^ developed a time-propagated MLWF calculation code in CP2K and applied it for the calculation of optical absorption spectra of periodic systems. They later extended their technique to electric quadrupole and magnetic dipole momenta to analyze ECD and ROA spectra of an aqueous solution of l/d-alanine dimer.^36^ This allows a sound calculation of ROA spectra with proper consideration of origin independence and provided the first ROA calculation including pre/on-resonance effects in a periodic setup.

Mattiat and Luber combined RT-TDDFT with AIMD to calculate nonresonance and resonance spectra only from the time domain.^166^ The resulting Raman excitation profile is given in Figure 20.

**Figure 20:**
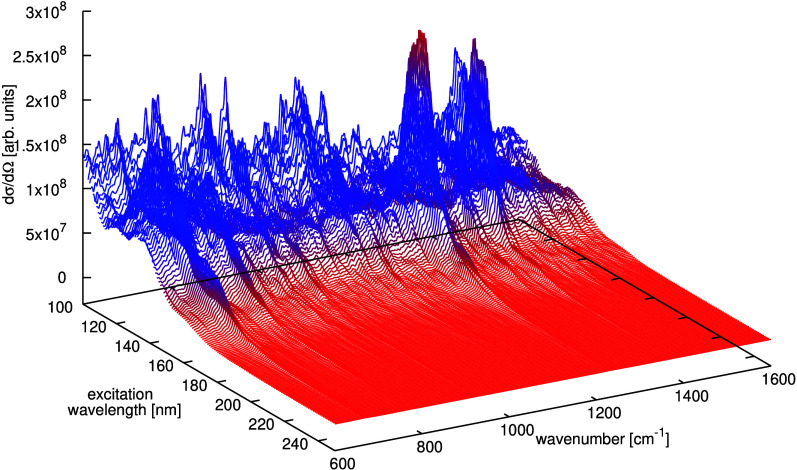
Raman excitation profile of liquid (*S*)-methyloxirane. Reproduced from ref 166. Copyright 2020 American Chemical Society.

##### Periodic Systems with **k**-Point Sampling

For periodic systems with **k**-point sampling, we adopted the VG formulation for RT-TDDFT calculations. In this formulation, a step function vector field **A**(*t*) = – *cF*_0_ **n**θ­(*t*), where **n** is the polarization vector, *F*_0_ is the amplitude, and θ­(*t*) is the Heaviside step function, is used to induce dynamics equivalent to that in an impulsive electric field **E**(*t*) = *F*_0_ **n**δ­(*t*) in the length-gauge formulation.

The density of **k**-point sampling is critically important to improve the accuracy of the spectrum of solids. In fixed-nuclei calculations, the full Brillouin zone can be reduced into the irreducible Brillouin zone by crystal symmetry and one can reduce the number of **k**-points without lowering accuracy. Details of the implementation of **k**-point reduction are shown in ref 241.

Figure 21 shows the dynamical conductivity of monolayer hexagonal boron nitride (h-BN) (lattice parameter *a* = 2.504 Å) calculated using LDA (PADE LDA^52^) and Goedecker–Tetter–Hutter (GTH) pseudopotential.^52^ The double-ζ basis set GTH-DZVP, as implemented in PySCF,^250^ was used as the basis set (the GTH-DZVP basis set is available also in CP2K, but there are differences in the orbital exponents between the CP2K and PySCF implementations). The **k** point was sampled using a 36 × 36 × 1 Monkhorst–Pack **k** mesh.^251^ The spectrum was calculated as ⟨**v**⟩_ω_ = ∫_0_^*T*^ d*t* e^iω*t*^e^–η^2^ *t* ^2^^⟨**v**⟩_*t*_ *f*(*t*), where *T* is the total simulation time, η is a line-broadening factor, and *f*(*t*) is a damping function inserted to reduce oscillation due to the finite-time cutoff. In this example, the line-broadening factor was set η = 0.1 eV, and an exponential damping function *f*(*t*) = e^–γ*t*^ with γ = 0.5 eV was applied. Figure 21 shows the dynamical conductivity σ­(ω) calculated using CP2K.

**Figure 21:**
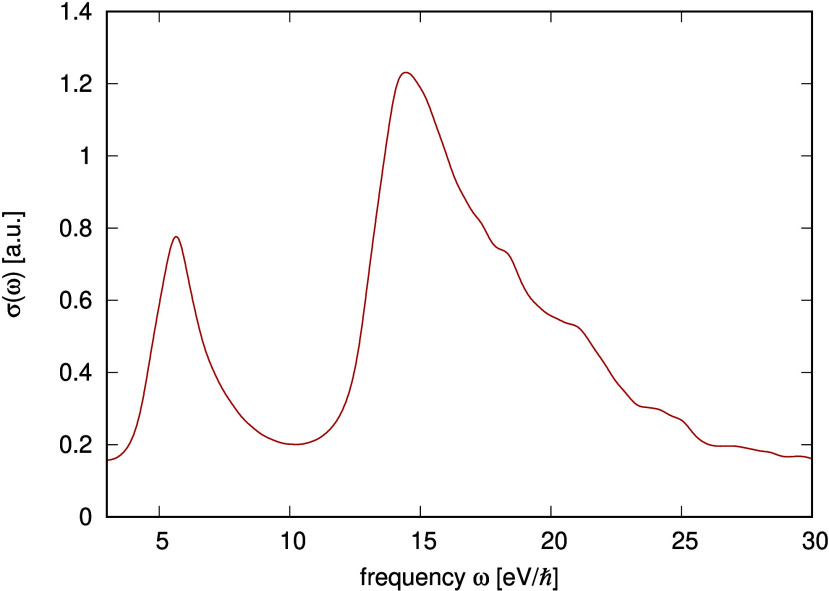
Dynamical conductivity σ­(ω) of monolayer h-BN.

#### 4.4 Section Summary

RT-TDDFT implementation for nonperiodic systems is equipped with multiple choices of perturbation and response with length and/or velocity gauge formulation, making the method a versatile tool to calculate different kinds of spectroscopic response functions by choosing the perturbation and response operators accordingly. In particular, RT-TDDFT was also applied to simulate vibrational spectra, by combining the method with a static normal mode based approach in the harmonic approximation, or with *ab initio* molecular dynamics to obtain full Raman and ROA excitation profiles. Also in periodic systems, transformation to MLWFs implemented for Γ-point calculations enables calculation of the local circulation part of the magnetic dipole moment and electric quadrupole moment. These implementations realize an exceptionally wide range of spectroscopic calculations, including (resonance) Raman, ECD, (resonance) ROA, etc. RT-TDDFT for periodic systems has recently been extended to **k**-point sampling calculations to realize more accurate and/or efficient calculations of solids. Challenges remain in further improving its accuracy. The accuracy of RT-TDDFT spectra depends on that of the KS Hamiltonian at each step, whereas the need for long-time simulation makes it difficult to apply advanced functionals such as (range-separated) hybrid functionals. This problem is pronounced in **k**-point sampling calculations of solids with strong electron correlations for which LDA or GGA gives qualitatively wrong results. Development of low-cost alternatives to hybrid functionals, such as DFT+U, is therefore critically important. The existing DFT+U implementation using empirical Hubbard parameters should preferably be equipped with ab initio calculation techniques^252–254^ for these parameters. A work is currently on the way to implement those techniques.

## Conclusions

The CP2K software package provides a powerful and versatile suite of DFT-based methods for studying excited states and spectroscopic properties of molecular and periodic systems. By implementing a range of complementary approaches—TD-DFPT, ΔSCF, DFPT, and RT-TDDFT—the CP2K software enables researchers to leverage the most appropriate technique for their specific application.

The TD-DFPT implementation^19,255,256^ in CP2K harnesses the combination of advanced computational techniques, such as the GPW and GAPW approach,^20^ the ADMM for efficient Fock exchange calculations,^63^ and semiempirical kernels through the sTDA.^64^ These methods allow TD-DFPT in CP2K to efficiently and accurately compute excited-state properties of complex systems. Furthermore, ongoing developments aim to further extend this framework by enabling spin-flip and mixed-reference spin-flip approaches, thereby broadening the accessible range of excited-state phenomena, particularly in open-shell systems.

The ΔSCF implementation in CP2K offers an efficient approach for calculating properties of specific excited states, particularly useful for large systems. Advanced algorithms like AIMOM, OT, and Switcher improve convergence and accuracy. Integration with subsystem density embedding methodology further enhances computational efficiency for periodic systems.

The NAMD capabilities implemented in CP2K represent an attractive tool for the study of photochemical processes and excited-state dynamics. The ΔSCF-based NAMD implementation has proven particularly valuable for investigating nonradiative deactivation mechanisms in the condensed phase such as liquids obeying PBC. Studies on systems such as diimide in water^23^ and cyclopropanone in aqueous solution^24^ have demonstrated the critical importance of explicitly accounting for solvent effects in excited-state dynamics. In addition to surface hopping approaches, Ehrenfest dynamics based on RT-TDDFT is also available.^25^ The ability to perform NAMD simulations with subsystem density embedding further enhances computational efficiency while maintaining accuracy, as shown in the comparative study of embedded versus nonembedded diimide systems.^115^

Complementing the ΔSCF approach, the TD-DFPT-based NAMD implementation in CP2K enables the study of systems in excited states. This capability can be enhanced by the inclusion of SOC effects, as has been showcased in the investigation of *o*- and *p*-nitrophenol photochemistry,^21^ where the inclusion of several singlet and triplet states was crucial for accurately describing the complex excited-state dynamics. The comparison between full TD-DFPT and QM/MM approaches in this study highlighted the importance of treating both solute and solvent at the same level of theory to capture phenomena such as intermolecular charge transfer states.

The DFPT implementation in CP2K enables the efficient calculation of various spectroscopic properties for gas and/or condensed-phase systems. This includes nonresonance Raman,^31–33,165–167^ infrared (IR) absorption,^38,42^ nonresonance Raman optical activity (ROA),^168,169^ sum frequency generation (SFG)^192^ and vibrational circular dichroism (VCD) spectra,^38,41–43,190^ as well as NMR^39,40^ and EPR^40^ properties. Nuclear velocity perturbation theory (NVPT)^41,45,190,257^ and magnetic field perturbation theory (MFPT)^41,45^ are also supported for the calculation of magneto-optical properties. Recent extensions employing atomic orbital-based response solvers promise improved scaling for large systems.^43^ The DFPT implementations have been successfully applied to study spectroscopic properties of complex systems, including liquids, solvated molecules, and surface-adsorbed species, where the efficient DFPT implementation combining static and dynamical (DFT+MD) methods have been employed to investigate the ROA, SFG, Raman, and IR spectra, demonstrating the versatility of DFPT capabilities.

RT-TDDFT provides a complementary approach well-suited for calculating optical spectra over a wide energy range. It supports nonperiodic and periodic systems (including **k**-point sampling). It can simulate, for example, UV–vis,^33^ electronic circular dichroism (ECD),^31,32^ Raman,^33^ and ROA^34,36^ spectroscopy. It allows for investigating the entire excitation profile including both static and dynamic effects and on-, pre-, off-resonance effects within one set of simulations. In periodic systems, propagated MLWFs realize in-depth analysis of subsystem-resolved spectra.^36^

The **k**-point sampling development^241^ extends its applicability to periodic materials, as shown for monolayer h-BN. Implementation of DFT+U^241^ further extends its applicability to strongly correlated systems at reasonable computational costs.

In summary, CP2K, like a “Swiss Army knife”, proves its versatility with its suite of excited-state and spectroscopic methods for gas- and condensed-phase (periodic) systems for static and dynamic calculations. These methods provide a powerful, unique toolkit for investigating a wide range of (photo)­physical and (photo)­chemical phenomena.

## Abbreviations

bpydp: 2,2′-bipyridine-4,4′-biphosphonic acid
AIMD: *ab initio* molecular dynamics
AIMOM: adapted initial maximum overlap method
AAT: atomic axial tensor
AO: atomic orbital
APT: atomic polar tensor
ADMM: auxiliary density matrix method
BCH: Baker–Campbell–Hausdorff
BP: Berry phase
BEC: Born effective charge
CP: circularly polarized
CIS: configuration interaction singles
CI: conical intersection
ΔSCF: delta self-consistent field
DFPT: density functional perturbation theory
DFT: density functional theory
DBCSR: distributed block compressed sparse row
ERI: electron repulsion integral
ECD: electronic circular dichroism
ETRS: enforced time reversible symmetry
xc: exchange–correlation
ESIPT: excited-state intramolecular proton transfer
FFT: fast Fourier transform
FS: fewest-switching
GIAO: gauge including atomic orbitals
GAPW: Gaussian and augmented plane wave
GPW: Gaussian and plane waves
GGA: generalized-gradient approximation
GTH: Goedecker–Teter–Hutter
h-BN: hexagonal boron nitride
HOMO: highest-energy occupied molecular orbital
HK: Hohenberg–Kohn
IR: infrared
ISC: intersystem crossing
KG: Kim–Gordon
KS: Kohn–Sham
LZ: Landau–Zener
LG: length gauge
TD-DFPT: linear-response time-dependent density functional perturbation theory
LDA: local-density approximation
LO: localized orbital
LUMO: lowest-energy unoccupied molecular orbital
MFPT: magnetic field perturbation theory
MLWF: maximally localized Wannier function
MOM: maximum overlap method
MD: molecular dynamics
MM: molecular mechanics
MO: molecular orbital
NA: nonadiabatic
NAC: nonadiabatic coupling
NAMD: nonadiabatic molecular dynamics
NE: nonembedded
NRD: nonradiative deactivation
NDPT: nuclear displacement perturbation theory
NVPT: nuclear velocity perturbation theory
OT: orbital transformation
2NP: *o*-nitrophenol
4NP: *p*-nitrophenol
PBC: periodic boundary conditions
PES: potential energy surface
QM: quantum mechanics
ROA: Raman optical activity
RPA: random phase approximation
RT-TDDFT: real-time time-dependent density functional theory
ROKS: restricted open-shell Kohn–Sham
RMSD: root-mean-square deviation
RS: rotational strength
SCF: self-consistent field
sTDA: simplified Tamm–Dancoff approximation
SOC: spin–orbit coupling
SP: spin purification
SDE: subsystem density embedding
TDA: Tamm–Dancoff approximation
TDDFT: time-dependent density functional theory
TDSE: time-dependent Schrödinger equation
TSH: trajectory surface hopping
TDM: transition dipole moment
TFS: Tully’s fewest-switch
UV: ultraviolet
UKS: unrestricted Kohn–Sham
VG: velocity gauge
VCD: vibrational circular dichroism
